# Earth System Model Analysis of How Astronomical Forcing Is Imprinted Onto the Marine Geological Record: The Role of the Inorganic (Carbonate) Carbon Cycle and Feedbacks

**DOI:** 10.1029/2020PA004090

**Published:** 2021-09-30

**Authors:** Pam Vervoort, Sandra Kirtland Turner, Fiona Rochholz, Andy Ridgwell

**Affiliations:** ^1^ Department of Earth and Planetary Science University of California Riverside CA USA; ^2^ MARUM Center for Marine and Environmental Sciences University of Bremen Bremen Germany; ^3^ Now at Department of Geography Research Group for Earth Observation Heidelberg University of Education Heidelberg Germany

**Keywords:** astronomical forcing, carbon cycling, early Cenozoic, earth system modeling, feedbacks, greenhouse climate

## Abstract

Astronomical cycles are strongly expressed in marine geological records, providing important insights into Earth system dynamics and an invaluable means of constructing age models. However, how various astronomical periods are filtered by the Earth system and the mechanisms by which carbon reservoirs and climate components respond, particularly in absence of dynamic ice sheets, is unclear. Using an Earth system model that includes feedbacks between climate, ocean circulation, and inorganic (carbonate) carbon cycling relevant to geological timescales, we systematically explore the impact of astronomically modulated insolation forcing and its expression in model variables most comparable to key paleoceanographic proxies (temperature, the δ^13^C of inorganic carbon, and sedimentary carbonate content). Temperature predominately responds to short and long eccentricity and is little influenced by the modeled carbon cycle feedbacks. In contrast, the cycling of nutrients and carbon in the ocean generates significant precession power in atmospheric CO_2_, benthic ocean δ^13^C, and sedimentary wt% CaCO_3_, while inclusion of marine sedimentary and weathering processes shifts power to the long eccentricity period. Our simulations produce reduced *p*CO_2_ and dissolved inorganic carbon (DIC) δ^13^C at long eccentricity maxima and, contrary to early Cenozoic marine records, CaCO_3_ preservation in the model is enhanced during eccentricity‐modulated warmth. Additionally, the magnitude of δ^13^C variability simulated in our model underestimates marine proxy records. These model‐data discrepancies hint at the possibility that the Paleogene silicate weathering feedback was weaker than modeled here and that additional organic carbon cycle feedbacks are necessary to explain the full response of the Earth system to astronomical forcing.

## Key Points

- Time‐varying astronomical forcing simulated on a multimillion‐year timescale using an Earth system model of intermediate complexity
- Strong 100 kyr power present in temperature and wt% CaCO_3_ is absent from δ^13^C cycles in the inorganic carbon reservoir
- Additional organic carbon feedbacks and a reduced weathering feedback likely played a role in early Cenozoic climate‐carbon cycle dynamics

## 1 Introduction

High‐resolution paleoclimate records across the Cenozoic Era show that global climate and carbon cycling are closely connected on astronomical timescales on the order of 10^4^–10^6^ years (e.g., Barnet et al., 2019; Mix et al., 1995; Pälike et al., 2006; Peterson et al., 2014; Sigman & Boyle, 2000; Turner et al., 2014; Wang et al., 2010; Westerhold et al., 2018; Zachos et al., 2001). In particular, during the early, ice‐free Cenozoic, benthic foraminiferal oxygen and carbon isotopes (δ^18^O and δ^13^C) show a strongly coherent astronomical influence (Cramer et al., 2003; Littler et al., 2014; Turner, 2014; Westerhold et al., 2011; Zachos et al., 2010), suggesting tightly coupled carbon cycle‐climate dynamics. Multiple records reveal periodic, astronomically paced negative δ^13^C excursions with a magnitude of 0.3‰–1.0‰ coinciding with deep water warming of 2°C–4°C as well as reductions in sedimentary calcium carbonate content (Lourens et al., 2005; Sexton et al., 2011; Zachos et al., 2010). Explanations for an astronomical trigger for these events, known as hyperthermals, invoke dynamics of a reduced carbon reservoir such as methane hydrates (Dickens, 2003; Lunt et al., 2011), permafrost (DeConto et al., 2012), wetlands (Kurtz et al., 2003; Zachos et al., 2010), or marine dissolved organic matter (Sexton et al., 2011), potentially in combination with thresholds in the climate system (Lunt et al., 2011).

Variations in global climate and the carbon cycle during the early, ice‐free Cenozoic, including “hyperthermals,” are predominately paced by short and long eccentricity cycles (Barnet et al., 2019; Lauretano et al., 2015; Littler et al., 2014; Turner, 2014; Westerhold et al., 2011, 2018; Zachos et al., 2001). While eccentricity is the only astronomical parameter that alters Earth's annual global mean solar energy insolation, the total variability is small (∼0.5 Wm^−2^) (Laskar et al., 2004). Local seasonal insolation forcing related to precession is much stronger (Berger, 1978), and therefore one hypothesis is that carbon‐climate variability is driven by precession forcing but modulated by eccentricity cycles (e.g., Maslin & Ridgwell, 2005; Zeebe et al., 2017). Additionally, the large mass of dissolved inorganic carbon in the ocean should act as a low‐pass filter on carbon cycle variability, producing muted precession signals relative to eccentricity (Cramer et al., 2003; Pälike et al., 2006).

Numerical models are key tools for testing hypotheses about the origin of astronomically forced climate‐carbon cycles. Previous studies have utilized atmosphere‐ocean global climate models (GCMs) to reveal the mechanisms by which the astronomical configuration exerts a significant control on surface dynamics. For instance, the GENESIS GCM (Thompson & Pollard, 1997) was used to evaluate Eocene (paleo)oceanographic differences between two precession configurations (Sloan and Huber, 2001). That same model (with a slab‐ocean) was used to reconstruct the extent of Eocene permafrost under various astronomical configurations, providing evidence for astronomically triggered carbon release at high latitudes (DeConto et al., 2012). Lunt et al. (2011) used the fully coupled HadCM3L GCM to show differences in ocean circulation under certain astronomical configuration, suggesting that variations in intermediate water mass temperature might drive the periodic destabilization of methane hydrates.

Because GCMs are computationally expensive, simulations usually apply fixed astronomical parameters, as in the studies described above, with the consequence that results cannot be compared directly to spectral analyses of proxy time series data. Exceptions include simulation of the complete deglacial transition with transient astronomical forcing (Liu et al., 2009), although this time interval is too short to capture complete astronomical cycles. Other studies have employed simpler box models to simulate the transient effects of astronomical forcing on the carbon cycle, typically using artificial forcing functions in the form of Eccentricity‐Tilt‐Precession (Laurin et al., 2015; Ma et al., 2011), or seasonal insolation curves (Pälike et al., 2006; Zeebe et al., 2017). While such models can simulate the transfer of spectral power amongst reservoirs and capture potential spectral shifts, the construction of the forcing functions requires basic presumptions about the mechanisms that drive the climate response.

Earth system Models of Intermediate Complexity (EMICs) bridge the gap between the most complex GCMs and box models that lack a dynamic ocean. Their computational efficiency allows generation of large ensembles of experiments and/or long integration time. EMIC studies of astronomical forcing, including those with the LOVECLIM (Goosse et al., 2010), CLIMBER‐2 (Ganopolski et al., 2001; Petoukhov et al., 2000), and UVic (Weaver et al., 2001) models, have focused on the Quaternary (Bounceur et al., 2015; Brovkin et al., 2002; Calov et al., 2005; Heinemann et al., 2014; Konijnendijk et al., 2011; Menviel et al., 2011; Xiao et al., 2013) or the Pliocene (Willeit et al., 2013). While these studies demonstrate the sensitivity of the climate and carbon cycle to insolation forcing, a modeling framework that systematically evaluates the imprint of astronomical forcing in various components of the carbon cycle is missing. Moreover, even these model simulations, with time‐varying orbital parameters, have been too short to evaluate the spectral power in model output on astronomical timescales (>10^5^ years). There remains a need to evaluate how astronomical forcing translates into the variables ultimately recorded by proxies (such as δ^18^O, δ^13^C, and CaCO_3_ content) on these timescales.

Here, we take a first step in this direction, driving the “cGENIE” EMIC (Ridgwell et al., 2007) with time‐varying astronomical forcing over multimillion‐year simulations. We develop and present a framework to assess the transient response of the Earth's climate‐carbon system to astronomical forcing under ice‐free greenhouse conditions, by separately quantifying the impact of insolation forcing on climate variability due to feedbacks in ocean circulation, marine productivity, CaCO_3_ compensation, and terrestrial weathering. The simulations presented here focus on feedbacks involving the marine inorganic (carbonate) carbon cycle. A follow‐up companion paper will separately interrogate the role of temperature‐dependent feedbacks in the marine organic matter cycle. Our experimental framework hence provides a systematic model evaluation of the propagation of spectral signals through the Earth system to facilitate comparison to proxy datasets.

## 2 Methods

The cGENIE model combines a dynamic 3D ocean model with a simplified 2D energy‐moisture‐balance atmosphere model (Edwards & Marsh, 2005). cGENIE includes a detailed representation of ocean biogeochemical cycles (Ridgwell et al., 2007) plus the long‐term (inorganic) carbon cycle represented through input and removal of dissolved carbon species via terrestrial rock weathering and calcium carbonate burial in marine sediments (Colbourn et al., 2013; Ridgwell & Hargreaves, 2007). This configuration of cGENIE has previously been employed on timescales up to 1 Myr to assess past climate events (e.g., Gutjahr et al., 2017) as well as the long‐term future carbon cycle response to fossil fuel carbon release (e.g., Lord et al., 2016; Winkelmann et al., 2015). Our methodological advance is to include spatiotemporal insolation forcing based on astronomical parameters using equations from Berger (1978). Parameters for eccentricity, obliquity, and precession (longitude of perihelion) are updated every 1,000 years and the daily mean insolation at each latitude is recalculated accordingly. We select parameters from the La2004 astronomical solution for an early Cenozoic time slice between 57 and 53 Ma (Laskar et al., 2004) and assume a solar constant of 1361.7 Wm^−2^, consistent with this interval.

### 2.1 Continental Configuration

**Figure 1:**
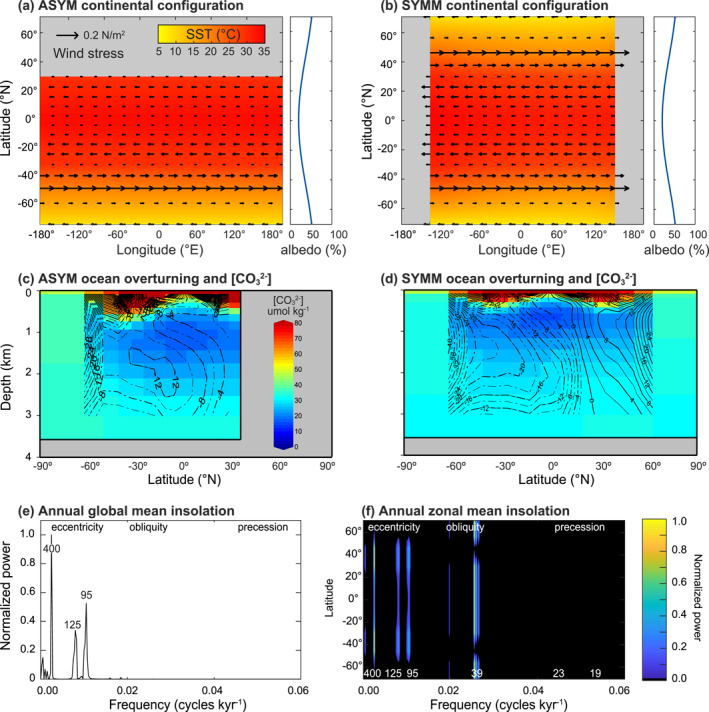
cGENIE configurations. (a and b) The continental landmass distribution (gray), prescribed wind stress fields (vectors), albedo profiles, and simulated sea surface temperature (SST, color coded) for (a) the asymmetric configuration (ASYM) and (b) symmetric configuration (SYMM). (c and d) The zonal mean ocean overturning strength indicated by contour lines, dashed for a counter‐clockwise circulation, solid lines for clockwise movement. Carbonate ion (CO_3_^2−^) concentrations for (c) ASYM and (d) SYMM are color coded. The bottom panels include normalized power spectra for (e) the annual global mean insolation and (f) the annual zonal mean insolation.

We employ a pair of deliberately idealized continental configurations (Figure 1) in order to distinguish climatic effects related to the carbon cycle from any astronomically forced effects that relate to the specific distribution of landmasses (e.g., Short et al., 1991). This provides a more generalized understanding of the ice‐free Earth system response to astronomical forcing than would have been possible, if we had chosen a specific continental configuration. However, it should be noted that it is not possible to understand all the ways in which paleogeography impacts atmosphere and ocean circulation and how these patterns may interact with astronomical forcing, using only a pair of end‐member configurations.

In the first configuration (symmetric configuration [SYMM]), a single continent spans pole to pole, creating a hemispherically symmetric landmass distribution (Vervoort et al., 2019). In the second, asymmetric configuration (ASYM), a single super‐continent covers the northern polar region. While no Cenozoic continental plate arrangement closely reflects either end‐member, we focus primarily on results using the ASYM setup because of the relative bias in the Northern Hemisphere landmass distribution throughout the Cenozoic. Comparable results for all SYMM experiments are included in the SI.

Because our model configuration lacks a dynamical atmosphere, a simplified zonally averaged wind‐stress profile is generated and applied to the ocean surface with matching wind products used in the atmospheric advection of heat and moisture and in calculating air‐sea gas exchange. Similarly, a meridional albedo profile is prescribed independent of the continental configuration but as a function of latitude only with the highest albedo values in the high latitudes, where the angle of incoming solar radiation enhances reflectivity. While differences in continental configuration should correspond to differences in albedo, our cGENIE experiments lack a land surface model to provide a more realistic pattern. Both SYMM and ASYM continental configurations consist of 18 × 18 equal‐area grids and 16 ocean depth levels of exponentially increasing thickness with depth. We truncate the vertical grid at 14 levels, giving a maximum depth of ∼3,575 m to approximate modern ocean volume and adopt a flat‐bottom bathymetry. We generate and apply a random spatial distribution of ocean sediment depths consistent with the modern hypsometric curve that is used in the pressure‐dependent calculation of calcium carbonate preservation. Boundary conditions were generated using the “muffingen” software suite v0.9.25 (https://doi.org/10.5281/zenodo.5130677) and are shown in Figure 1 and S1.

### 2.2 cGENIE Spin‐Up Stages

The model is equilibrated in a two‐step spin‐up process for each of the SYMM and ASYM configurations. An initial 20,000‐year spin‐up equilibrates ocean dynamics and ocean biogeochemical cycling with atmospheric CO_2_ concentrations fixed at 834 ppm (Ridgwell & Schmidt, 2010), emulating an early Cenozoic “warm climate” and yielding sufficiently high temperatures to prevent both perennial and seasonal sea ice formation at the high latitudes. Values for the astronomical parameters are fixed during spin‐up at their average values across the modeled interval (53–57 Ma). The initial values of mean ocean alkalinity (ALK), dissolved inorganic carbon (DIC), nutrient (phosphate) concentrations, and major ion concentrations of Ca^2+^, Mg^2+^, and SO_4_^2−^ of the model follows previous Paleocene cGENIE configurations (e.g., Ridgwell & Schmidt, 2010).

In the second stage spin‐up, also with fixed astronomical parameters, the long‐term carbon cycle is enabled by allowing loss of solutes from the ocean through marine CaCO_3_ burial and balancing this with a terrestrial weathering flux. Atmospheric CO_2_ is stabilized close to 834 ppm by adjusting the balance between volcanic outgassing and terrestrial silicate weathering (dependent on land temperature as described in Colbourn et al., 2013 and Lord et al., 2016). The final steady states of ocean carbonate chemistry differ slightly between SYMM and ASYM, a consequence of the differences in continental distribution and hence large‐scale pattern of ocean circulation (Figures 1c and 1d, Table S1).

### 2.3 Experimental Design

Starting from these initial steady states, the effects of astronomical forcing on climate and the carbon cycle are systematically investigated, running cGENIE transiently for 4 million years to allow the evaluation of astronomical cycles with periods up to 500 kyr. We conduct a set of five simulations for both SYMM and ASYM in which the complexity of the carbon cycle is sequentially increased (Table 1).

**Table 1:** Experimental Design

	Climate‐CO_2_ feedback	Marine export feedback	CaCO_3_ compensation	Terrestrial weathering
Exp.0	‐	‐	‐	Tracking
Exp.1	✓	‐	‐	Tracking
Exp.2	✓	✓	‐	Tracking
Exp.3	✓	✓	✓	Fixed
Exp.4	✓	✓	✓	Responsive

*Note*. For Exp.0–4, shown which carbon‐climate feedbacks are excluded (‐) and included (✓). All experiments are performed under SYMM (hemispherically symmetric continent) and ASYM (supercontinent at the Northern Hemisphere) configurations. Note that marine surface productivity is present, but unresponsive to astronomical forcing in Exp.0 and 1 while surface productivity varies with astronomical forcing in Exp.2–4. For terrestrial weathering, ‘tracking’ indicates that weathering fluxes are exactly equal to (variable) CaCO_3_ burial fluxes, ‘fixed’ means weathering fluxes are fixed and decoupled from CaCO_3_ burial, “responsive” indicates weathering rates vary in response to changes in land temperature.

In the simplest experiment (Exp.0), we disable all carbon cycle feedbacks and fix radiative forcing with respect to an atmospheric CO_2_ composition of 828 ppm for ASYM and 834 ppm for SYMM (Table S1). Variability in global climate is thus only a function of the temporal radiative balance and feedbacks relating to physical processes, such as meridional ocean and atmospheric heat transport, and ocean‐atmosphere exchange of heat and moisture. Cycles produced in atmospheric CO_2_ have no influence on the modeled temperature variability.

In the subsequent experiment (Exp.1), we allow variations in atmospheric CO_2_ to modulate surface temperature. The magnitude and spatial distribution of export production of both particulate organic carbon (POC) and CaCO_3_ is fixed, and weathering fluxes are forced to exactly track CaCO_3_ burial such that the ocean ALK and calcium ion inventories become invariant. Global climate now additionally responds to carbon cycle feedbacks related to the controls of ocean circulation and solubility on ocean CO_2_ uptake. Resultant changes in ocean carbonate chemistry will impact the preservation of CaCO_3_ (but does not cause any change in ocean ALK and calcium ion inventories) plus the distribution of DIC δ^13^C. With fixed export, preservation of CaCO_3_ in surface sediments varies as a function of temperature, salinity, and (CO_3_^2−^) of overlying seawater. δ^13^C of DIC is controlled by multiple fractionation processes. First, isotopic partitioning occurs during re‐equilibration between CO_2_, HCO_3_^−^, and CO_3_^2−^ (Ridgwell et al., 2007; Zeebe & Wolf‐Gladrow, 2001). A second temperature‐dependent isotopic fractionation occurs during the air‐sea gas exchange (Ridgwell et al., 2007; Zhang et al., 1995). We also model the fractionation associated with organic matter formation as a function of aqueous CO_2_ concentration (Rau et al., 1996; Ridgwell, 2001) as well as temperature‐dependent isotopic fractionation between (HCO_3_^−^) and CaCO_3_ (Mook, 1986). See Turner and Ridgwell (2016) for a detailed description of the representation of δ^13^C in cGENIE including evaluation of simulated spatial patterns.

In Exp.2, POC (with CaCO_3_ in a fixed ratio of 0.2 mol:mol) export is now allowed to vary in space and time as a function of light and nutrient (phosphate) availability following Ridgwell et al. (2007). Exported POC is remineralized through the water column according to a fixed exponential decay depth profile (Ridgwell et al., 2007). Again, all carbon, ALK, and calcium removed from the system through CaCO_3_ burial is automatically replenished with an equal flux from terrestrial weathering. This configuration provides ocean‐atmosphere carbon cycle feedbacks but no geological carbon feedbacks.

We add the first geological carbon feedback in Exp.3 by decoupling terrestrial weathering (providing the alkaline building blocks for CaCO_3_ production) from marine CaCO_3_ burial. Weathering rates are fixed at the values obtained from the end of the open system spin‐up, while CaCO_3_ preservation varies based on CaCO_3_ export productivity and the saturation state of overlying water. The ocean ALK (and Ca^2+^) inventory can now vary and impact *p*CO_2_ via ‘carbonate compensation’.

Finally, Exp.4 completes the representation of the long‐term (inorganic) carbon cycle by including a terrestrial weathering feedback (see Lord et al., 2016 for an analysis of the modes of response and characteristic timescales of the complete system). Carbonate weathering varies linearly with mean global air temperature over land whereas silicate weathering responds exponentially (Colbourn et al., 2013).

To elucidate the role of each set of carbon cycle feedbacks in the Earth system response to astronomical forcing, we investigate the difference between each set of experiments, for example, output of Exp.3 is subtracted from output of Exp.4 to isolate the impact of the astronomically forced weathering feedback on the Earth system. Hence while Figures 3d–3f presents the response in *p*CO_2_, benthic DIC δ^13^C, and wt% CaCO_3_ that results from the astronomically forced changes in ocean circulation and the solubility feedback (Exp.1), subsequent Figures 4d–4f through 6d–6f) are shown in the form of anomaly plots for the difference from the previous experiment. Likewise, we plot the change in the power spectra (ΔFFT) with respect to the previous experiment to demonstrate the relative weakening (negative power) or amplification (positive power) of astronomical cycles when adding feedbacks.

**Figure 2:**
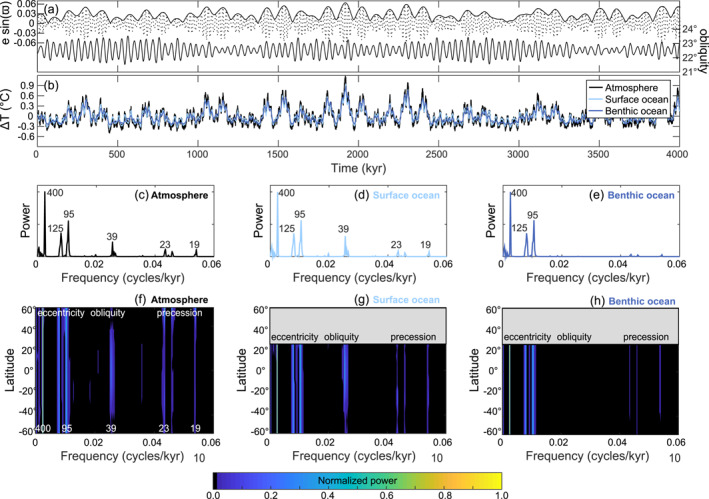
Astronomical climate evolution in asymmetric configuration (ASYM) of Exp.0. (a) The eccentricity, obliquity, and the precession index (*e* sin *ϖ*) used to calculate the daily mean insolation across 4 million years of cGENIE simulation. (b) The simulated change in the global annual mean temperature in the atmosphere (black), surface (light blue), and benthic ocean (dark blue) with their respective normalized power spectra in panels (c–e) calculated using the Fast Fourier Transform. Each value near a spectral peak indicates the associated period of the cycle in kyr. The power spectra of temperature in the (f) atmosphere, (g) surface, and (h) deep ocean are shown for each latitude.

### 2.4 Timeseries Analysis

Spectral analysis was performed on model output variables with a simple Fast Fourier Transform (FFT) to extract the dominant cycles (shorter than 500 kyr) and their average ranges. The average range is equal to twice the average amplitude across the 4 million year interval for each spectral peak determined by the FFT. Because model output is (white) noise‐free in the absence of internal interannual variability in cGENIE, further analysis to determine the statistical significance of the astronomical peaks is not required. The phasing between the imposed short and long eccentricity forcing and cycles in model variables of interest are determined using a multi‐taper coherence method (Table S2). Because model output is saved at 1000‐year intervals over the 4 Myr long experiments, we are unable to diagnose any lags with a duration less than 1,000 years.

## 3 Results and Discussion

In the following sections we describe the main results of Exp. 0–4, separately assessing the role of specific carbon cycle feedbacks in the Earth system response to astronomical forcing in proxy relevant model outputs, including ocean temperature, atmospheric pCO_2_, DIC δ^13^C, and sedimentary wt% CaCO_3_. We focus on mean annual output and evaluate signals in terms of the total variability generated, distribution of spectra power, the average range of individual cycles across the 4 million year interval, and phasing with respect to astronomical forcing and between model output. Results of the asymmetric (ASYM) world are presented in the main figures, while comparison with the equivalent SYMM simulations is provided in the final paragraph of each subsection. Corresponding graphical SYMM analyses are provided as Figures S2–S8. See Table S2 for a summary of cycle ranges and phasing calculated for each experiment. Comparison between ASYM and SYMM results allows us to evaluate how latitudinal heterogeneity in the landmass distribution impacts the global response to astronomical forcing. Figure 1 shows the power spectra of mean annual insolation as a global mean (Figure 1e) and with latitude (Figure 1f). Annual global mean insolation power spectra is dominated by eccentricity, while for most latitudes, obliquity dominates mean annual power spectra. Precession only appears significantly in seasonal insolation power spectra.

### 3.1 Global Climate Response

In Exp.0 (Figure 2), annual global mean air temperature responds to annual global mean insolation forcing but with no CO_2_ feedback on climate. In the ASYM configuration, the maximum variability in atmospheric temperature in response to astronomical forcing is 1.7°C. Spectral power in the global mean annual air temperature is dominated by the 400 kyr (long eccentricity) and 125 and 95 kyr (short eccentricity) periods (Figure 2c). The distribution of spectral power in global mean annual air temperature is similar to the distribution of spectral power in mean annual temperature at individual latitudes (Figure 2f). Seasonal averaging removes power in Earth's mean annual temperature response at higher frequencies, and local mean annual temperature does not respond linearly to the local mean annual insolation (contrast Figure 1f with Figure 2f). Mean annual insolation at most latitudes is dominated by obliquity power (Figure 1f), and obliquity power also appears in global mean annual air temperatures. In ASYM simulations, small precession cycles in the global mean annual air temperature result from the different heat capacity of each hemisphere and are consistent with the dominance of precession power in mean annual air temperature over the modeled Northern Hemisphere (NH) landmass (also recognized by Short et al., 1991) (Figure 2f). The NH, where the continent is located, has a smaller heat capacity than the Southern Hemisphere (SH) and thus the increase in the global annual mean temperature is greater when the NH is tilted toward the Sun at perihelion, compared to when the SH is tilted toward the Sun at perihelion.

Changes in the surface air temperature are instantaneously transferred (with a lag less than the 1 kyr interval of model data saving) to the surface ocean. Although the maximum variability in global mean annual sea surface temperature (SST) is slightly smaller than global mean annual surface air temperature (1.3°C vs. 1.7°C), the distribution of spectral power is nearly identical (Figures 2c and 2d). Astronomically forced temperature changes in the benthic ocean are slightly reduced compared to the surface ocean, with a maximum variability of 1.1°C and spectral power is dominated by eccentricity (Figure 2e) with respective average ranges of 0.25°C and 0.30°C for the short and long eccentricity cycles. The major difference between the SST and benthic ocean temperature power spectra is a loss of power at obliquity and precession frequencies. On a global mean, benthic ocean temperature lags eccentricity forcing by ∼1 kyr, consistent with the timescale of ocean overturning.

Astronomically forced climate evolution in SYMM is comparable to ASYM in Exp.0 with the exception that the maximum variability in temperature is slightly reduced and precession cycles are absent (Figure S2). We attribute the loss of precession power to the similar heat capacity of both hemispheres in SYMM.

### 3.2 Ocean Solubility Pump

**Figure 3:**
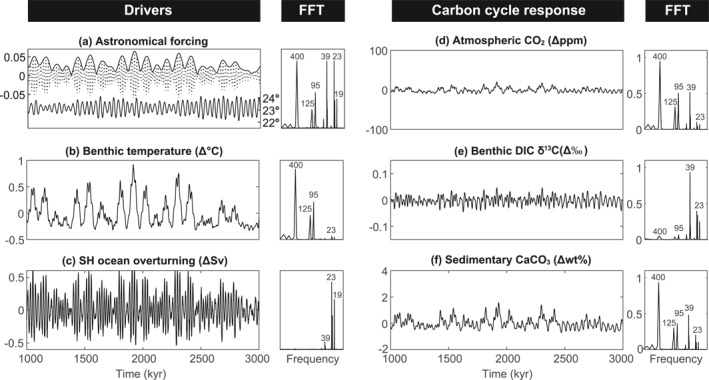
Exp.1, ocean circulation and CO_2_ solubility feedbacks in asymmetric configuration (ASYM). (a) Astronomical forcing parameters (eccentricity, obliquity (in degrees), and precession index (e sin *ϖ*)) and their Fast Fourier Transform (FFT) normalized to the highest individual power. (b) Change in benthic ocean temperature. (c) Change in the maximum Southern Hemisphere ocean overturning strength, defined by the maximum overturning strength reached in the SH across all ocean depth levels. (d) Change in atmospheric CO_2_. (e) Change in benthic δ^13^C of dissolved inorganic carbon (DIC). (f) Change in wt% CaCO_3_. All variables are annual global mean values and are accompanied by their respective FFTs, normalized to the peak with maximum power.

Astronomical forcing induces spatiotemporal variations in ocean temperature (Figure 2), and alters the strength of ocean overturning circulation. By enabling a CO_2_‐climate feedback in Exp.1 (Figure 3), changes in atmospheric *p*CO_2_ resulting from the influence of solubility and ocean circulation on ocean carbon uptake can now modulate the direct temperature response to astronomical forcing (cf. Exp.0).

The power spectrum of modeled global ocean overturning is dominated by precession (Figure 3c). Ocean overturning is a seasonal process driven by maximum deep‐water formation close after the coldest months at latitudes where seasonal insolation is dominated by precession power. Precession periodically facilitates relatively more intense overturning when the SH winter occurs at aphelion compared to perihelion. Enhanced deep‐water formation and increased ventilation of the ocean interior physically transports respired carbon back to the surface ocean, and thereby slightly increases atmospheric *p*CO_2_. However, the relatively small changes in the strength of ocean overturning (maximum variability of ∼1 Sv, corresponding to a change in the maximum ocean ventilation age of ∼100 years) are insufficient to drive major redistributions of carbon between the ocean and atmosphere. The average range of precession cycles in *p*CO_2_ is <3 ppm (Figure 3d).

The *p*CO_2_ power spectra is instead dominated by long eccentricity with a significant contribution of power at short eccentricity and obliquity. The total variability is ∼32 ppm with average ranges of obliquity and short eccentricity cycles in *p*CO_2_ of ∼5 ppm and ∼6 ppm for long eccentricity cycles, resulting from feedbacks due to ocean solubility and carbon uptake. More CO_2_ is released from the surface ocean into the atmosphere at eccentricity maxima when global annual mean temperatures are highest (Figure 3d). However, CO_2_ uptake is not spatially or seasonally uniform and is most significant at cold months in the high latitudes, resulting in obliquity cycles with a range comparable to eccentricity cycles.

The global mean benthic DIC δ^13^C power spectrum shows maximum variability of 0.09‰. Obliquity and precession dominate the power spectrum with very little power at eccentricity (Figure 3e). The lack of eccentricity power in δ^13^C timeseries demonstrates that the local and seasonal changes in temperature are particularly significant controls on the modeled fractionation processes. Precession power is consistent with the impact of ocean overturning on the physical transport of low‐δ^13^C remineralized carbon from the deep to the surface ocean, generating cycles with an average range of ∼0.02‰ that are anti‐phased between the surface and deep ocean (Figure 3e). Obliquity power arises primarily due to changes in fractionation associated with export production and generates cycles with a comparable average range of ∼0.02‰ also anti‐phased with surface ocean DIC δ^13^C cycles. Reduced fractionation in the surface ocean leads to isotopically lighter surface DIC while relatively heavier carbon respires at depth which increases the benthic DIC δ^13^C. Although the magnitude and spatial patterns of export are fixed, fractionation in the formation of organic matter depends on the concentration of aqueous CO_2_ and fractionation in the formation of calcite is temperature‐dependent, both of which drive changes in δ^13^C in response to obliquity‐paced changes in temperature and air‐sea gas exchange.

The power spectrum for global mean marine sedimentary wt% CaCO_3_ closely resembles that of atmospheric CO_2_, but with slightly amplified obliquity and precession power (Figures 3d and 3f). Strong eccentricity power in wt% CaCO_3_ is consistent with the dominance of eccentricity on deep ocean temperature (Figure 3b). Higher temperature at eccentricity maxima increases Ω_calcite_ and lowers CaCO_3_ solubility, promoting CaCO_3_ preservation. Precession and obliquity cycles are consistent with the influence of ocean circulation and solubility‐driven changes in CO_2_ uptake on ocean chemistry. Changes in benthic ocean (CO_3_^2−^), which also drive changes in Ω_calcite_, are dominated by precession and obliquity (Figure S9a).

The relative loss of high frequency power in wt% CaCO_3_ compared to benthic ocean (CO_3_^2−^) is also a function of calculating wt% CaCO_3_ from the sedimentary mixed layer, that is, the upper ca. 5 cm of the sediment column in which mixing homogenizes the solid composition (Ridgwell, 2007a, 2007b). This mixing acts as a low‐pass filter, amplifying eccentricity‐driven cycles in wt% CaCO_3_ arising from temperature‐dependency relative to the precession and obliquity cycles from (CO_3_^2−^)‐dependency. Averaging sedimentary content over the upper 5‐cm also induces a lag between 100 and 400 kyr eccentricity forcing and the global wt% CaCO_3_ of 8 kyr (29°) and 9 kyr (7°), respectively.

Including solubility‐related carbon feedbacks in the SYMM Exp.1 simulation results in similar power spectra compared to ASYM, despite the relative symmetry of ocean overturning (Figures 1c and 1d). Ocean overturning in SYMM is also primarily paced by precession (Figure S3c), creating distinct ∼20 kyr cycles in benthic DIC δ^13^C (Figure S3e). However, precession power is absent in global mean wt% CaCO_3_ (Figure S3f). Astronomically forced changes in ocean chemistry largely cancel out on precession timescales because the NH and SH overturning operate with roughly comparable strengths and in consequence, benthic ocean (CO_3_^2−^) is dominated by eccentricity and absent of precession power (Figure S9b). The lag between 100 and 400 kyr eccentricity forcing and global CaCO_3_ wt% in SYMM is 7 kyr.

### 3.3 Marine Surface Productivity

**Figure 4:**
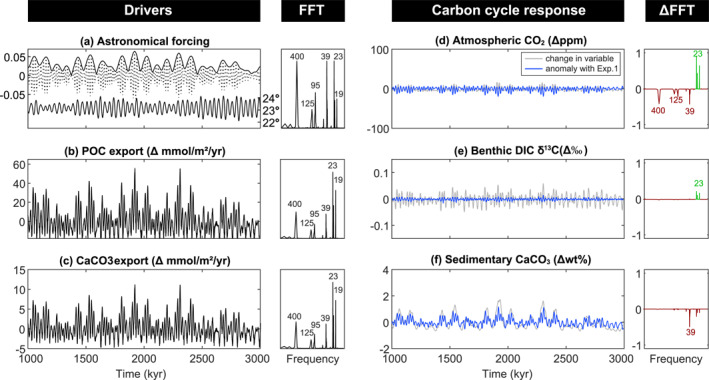
Exp.2, marine surface productivity feedback in asymmetric configuration (ASYM). (a) Astronomical forcing parameters (as per Figure 3a). (b) Change in the export of particulate organic carbon. (POC). (c) Change in the export of CaCO_3_. (d) Change in atmospheric CO_2_. (e) Change in benthic δ^13^C of dissolved inorganic carbon (DIC). (f) Change in wt% CaCO_3_. All variables are annual global mean values and are accompanied by their respective Fast Fourier Transforms (FFTs), normalized to the peak with maximum power. In blue, the values are plotted as anomalies with the previous experiment and depict the change driven by the marine productivity feedback only. Likewise, the FFTs are plotted as anomalies. A positive (green) value on the *y*‐axis indicates an increase in relative power and a negative (red) value indicates a decrease in relative power compared to the previous experiment.

Exp.2 elucidates how feedbacks involving marine productivity modifies the Earth system response to astronomical forcing. Global mean POC export production in ASYM (Figure 4b) is dominated by precession, illustrating the influence of changes in ocean circulation on surface nutrient supply and consistent with the seasonality of production. POC export also shows eccentricity modulation because elevated global ocean temperatures at eccentricity maxima result in stronger ocean overturning (Figure 3c), and elevated surface nutrient availability. CaCO_3_ export shows the same power spectrum as POC export because of the use of a fixed rain ratio (Figure 4c).

Relative to Exp.1, precession cycles in *p*CO_2_ become more pronounced at the expense of eccentricity cycles (Figure 4d) which reduces the total *p*CO_2_ variability to ∼26 ppm. Increased precessional power is a direct consequence of the impact that production and export of organic matter and carbonate has on surface DIC and ALK. The net impact is a decrease in mean annual surface ocean CO_2_[aq] of 0.7% (or 0.173 μmol/kg) corresponding to maximum surface productivity. This translates roughly to a 0.7% reduction in *p*CO_2_, equating to precession cycles with an average range of ∼6 ppm, double the magnitude from Exp.1.

Changes in the magnitude and spatial pattern of export production do not notably change the power spectrum of benthic DIC δ^13^C compared to Exp.1 except for a slight increase in precession power (Figure 4e). The similarity in the carbon isotope response of Exp.1 and Exp.2 is because Exp.1 already incorporated the astronomically driven changes in isotope fractionation associated with export production as described in Section 3.1.

Sedimentary wt% CaCO_3_ shows a slight loss of higher frequency power along with an increase in the maximum variability (Figure 4f). This shift in the power spectrum contrasts with the response of atmospheric *p*CO_2_ and benthic δ^13^C to the addition of varying export production. As CaCO_3_ export increases in response to seasonal precession and obliquity forcing, surface and deep ocean [CO_3_^2−^] decreases, lowing Ω_calcite_ and promoting CaCO_3_ dissolution. Hence, even though CaCO_3_ export and rain to the seafloor increases, the lower [CO_3_^2−^] prevents further CaCO_3_ preservation. On eccentricity timecales, CaCO_3_ export and benthic [CO_3_^2−^] vary in‐phase and amplify short and long eccentricity power. The relative phasing between eccentricity forcing and CaCO_3_ is unaffected by the responsive export productivity.

Global mean export production in SYMM Exp.2 (Figure S4c) varies less than ASYM Exp.2 (Figure 4c). In ASYM, the impact of precession is amplified because most of the export productivity occurs in the SH. Hemispheric productivity in SYMM is roughly balanced, which cancels out precession‐induced changes and leaves eccentricity to dominate power spectra of atmospheric *p*CO_2_. Variations in annual global mean *p*CO_2_, benthic DIC δ^13^C, and CaCO_3_ burial are muted, as a result of this hemispheric cancelation and there is little change with respect to Exp.1 (Figure S4d–S4f).

### 3.4 Carbonate Compensation

We evaluate the impact of the carbonate compensation feedback in modifying the response to astronomical forcing in Exp.3 by now allowing burial of CaCO_3_ to modify the global ocean ALK inventory while no longer forcing weathering fluxes to always track burial, and contrast these results with Exp.2.

**Figure 5:**
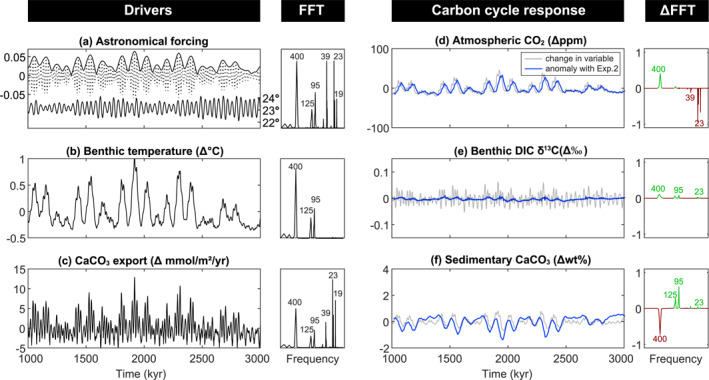
Exp.3, deep marine CaCO_3_ feedback in asymmetric configuration (ASYM). (a) Astronomical forcing parameters (as per Figure 3a). (b) Change in benthic ocean temperature. (c) Change in the export of CaCO_3_. (d) Change in atmospheric CO_2_. (e) Change in benthic δ^13^C of dissolved inorganic carbon (DIC). (f) Change in wt% CaCO_3_. All variables are annual global mean values and are accompanied by their respective Fast Fourier Transforms (FFTs), normalized to the peak with maximum power. In blue, the values are plotted as anomalies with the previous experiment and depict the change driven by the marine CaCO_3_ feedback only. Likewise, the FFTs are plotted as anomalies. A positive (green) value on the *y*‐axis indicates an increase in relative power and a negative (red) value indicates a decrease in relative power compared to the previous experiment.

The total variability in atmospheric *p*CO_2_ increases to 64 ppm in Exp.3 and is relatively elevated during eccentricity maxima compared to Exp.2. The burial‐weathering imbalance reduces the ocean carbon uptake capacity and this feedback impacts atmospheric *p*CO_2_ more than enhanced productivity. Thus, *p*CO_2_ shows enhanced eccentricity power and reduced power at high frequencies relative to Exp.2 (Figure 5d). Eccentricity cycles are significantly increased compared to Exp.2, with an average cycle range of almost 20 ppm for long eccentricity.

Consistent with *p*CO_2_, power in benthic δ^13^C becomes slightly amplified at eccentricity frequencies in Exp.3 relative to Exp.2. The average range of long eccentricity cycles increases from 0.006‰ to ∼0.011‰. Yet, obliquity and precession still remain the dominant cycles and control the total variability in DIC δ^13^C of 0.11‰.

Compared to Exp.0–2, increased CaCO_3_ burial now removes ALK that is not resupplied by weathering (which is fixed), leading to lower deep ocean Ω_calcite_ that counteracts increased burial. This results in reduced 100 and 400 kyr wt% CaCO_3_ cycles in Exp.3 compared to Exp.2. The wt% reduction in 400 kyr cycles is much larger (from 0.7 to 0.2 wt%) than that of 100 kyr cycles (from 0.5 to 0.4 wt%) because the CaCO_3_‐weathering imbalance is sustained over a much longer period that exceeds the residence time of carbon in the ocean, shifting the dominant spectral power from the 400 kyr to the 100 kyr eccentricity cycles (Figure 5f). The wt% CaCO_3_ is now leading short eccentricity forcing by 5 kyr.

The change in the CaCO_3_ preservation cycles in SYMM (Figure S5) are similar to ASYM because the impact of the ocean ALK imbalance on CaCO_3_ at eccentricity maxima is similar. In SYMM too, this feedback amplifies 100 and 400 kyr cycles in *p*CO_2_ with the 400 kyr cycle producing the greatest increase in the average range. The long eccentricity remains the dominant *p*CO_2_ cycle with a value normalized to 1, and therefore does not show up as an increase in the ΔFFT plot, contrary to ASYM in which dominant power shifted from precession to long eccentricity. The long eccentricity cycle in δ^13^C increases in average range from 0.002‰ to 0.003‰ and is considered negligible.

### 3.5 Terrestrial Weathering Feedback

In our final experiment (Exp.4), we employ a temperature‐dependent weathering parameterization (Colbourn et al., 2013) as opposed to keeping weathering rates invariant (Exp.3) or always balancing burial with weathering (Exp.0‐2). As air temperatures over land rise, terrestrial silicate and carbonate weathering accelerate and thereby increase the transport of dissolved carbon and ALK to the surface ocean, allowing for greater carbon uptake and enhanced sedimentary CaCO_3_ preservation.

**Figure 6:**
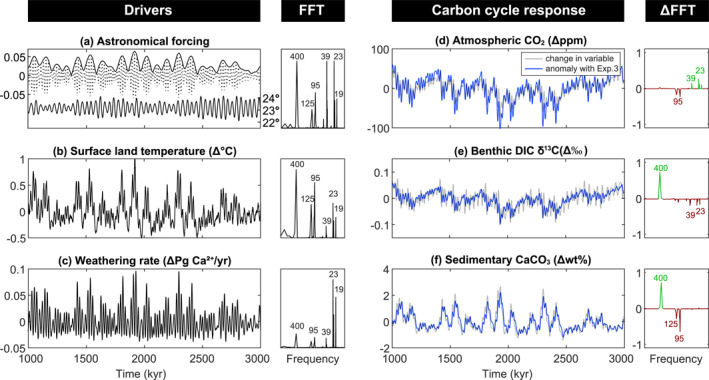
Exp.4, terrestrial rock weathering feedback in asymmetric configuration (ASYM). (a) Astronomical forcing parameters (as per Figure 3a). (b) Change in surface land temperature. (c) Change in the rate of terrestrial weathering. (d) Change in atmospheric CO_2_. (e) Change in benthic δ^13^C of dissolved inorganic carbon (DIC). (f) Change in wt% CaCO_3_. All variables are annual global mean values and are accompanied by their respective Fast Fourier Transforms (FFTs), normalized to the peak with maximum power. In blue, the values are plotted as anomalies with the previous experiment and depict the change driven by the terrestrial weathering feedback only. Likewise, the FFTs are plotted as anomalies. A positive (green) value on the *y*‐axis indicates an increase in relative power and a negative (red) value indicates a decrease in relative power compared to the previous experiment.

The power spectrum of annual global mean surface land temperature is dominated by 100 and 400 kyr eccentricity (Figure 6b). The relatively amplified precession power in land temperature compared to atmospheric temperature is a consequence of the asymmetric landmass distribution in ASYM. Land temperatures, and therefore global weathering rates, are elevated when the NH is tilted maximally toward the Sun. Weathering rates consequently are dominated by precession cycles (Figure 6c).

Precession power in the weathering rate generates precession cycles in atmospheric *p*CO_2_ with an average range of 17 ppm, significantly larger compared to Exp.3. The average range of 400 kyr CO_2_ cycles increases in comparison to Exp.3 to a value of 26 ppm. This relative increase in 400 kyr power is not apparent in the ΔFFT plot (Figure 6d) because the power spectra are normalized, meaning that the dominant cycle (long eccentricity) maintains a value of 1 for Exp.3. The dominance of long eccentricity on atmospheric CO_2_ is a result of the long ocean‐atmosphere carbon residence time due to relatively slow weathering rates. The slow response time of the ocean's carbon reservoir acts as a low‐pass filter (Cramer et al., 2003; Laurin et al., 2015; Pälike et al., 2006), further demonstrated by large phase differences of 112° and 125° between *p*CO_2_ and the short and long eccentricity forcing, respectively, that did not exist before. The total variability modeled in *p*CO_2_ increases to 132 ppm.

Maximum variability of 0.19‰ in benthic DIC δ^13^C arises from the imbalance between weathering δ^13^C and the δ^13^C of buried CaCO_3_ (Figure 6e). Benthic DIC δ^13^C declines in response to elevated weathering and carbonate burial at long eccentricity maxima because relatively heavy carbon (δ^13^C_CaCO3_ = δ^13^C_DIC_ + 1.9‰) is removed from the ocean through the burial of CaCO_3_. Burial of carbonate thus dominates the response of benthic DIC δ^13^C despite the input of high δ^13^C via weathering at eccentricity maxima. A high δ^13^C value for weathered carbonate is required in our model framework to balance volcanic carbon input because we do not simulate organic carbon burial. Inclusion of organic carbon burial would allow us to use a δ^13^C of weathered CaCO_3_ more comparable to the δ^13^C of marine CaCO_3_. However, because the response of benthic DIC δ^13^C is negative at long eccentricity maxima, a lower value for the δ^13^C of weathered carbon would mean that weathering less effectively counteracts the impact on benthic DIC δ^13^C of increasing CaCO_3_ burial and would drive an even greater decline in DIC δ^13^C.

Sedimentary wt% CaCO_3_ also shows a relative increase in power at the long eccentricity frequency and greater phase lag with eccentricity forcing, consistent with the slow weathering feedback. The wt% CaCO_3_ lags the short eccentricity forcing by 10 kyr.

The primary difference in SYMM simulations is the lack of precession power in the weathering rate and consequently in *p*CO_2_, benthic DIC δ^13^C, and global mean wt% CaCO_3._ With landmasses distributed evenly, both the NH and SH have an equal amount of terrestrial area available for weathering, which minimizes the temporary imbalance on precessional timescales. Power spectra still show the relative shift toward long eccentricity forcing driven by the low pass filtering effect of the ocean carbon reservoir, consistent with ASYM (Figure S6).

### 3.6 Astronomical Response to (Inorganic) Carbon Cycle Feedbacks

**Figure 7:**
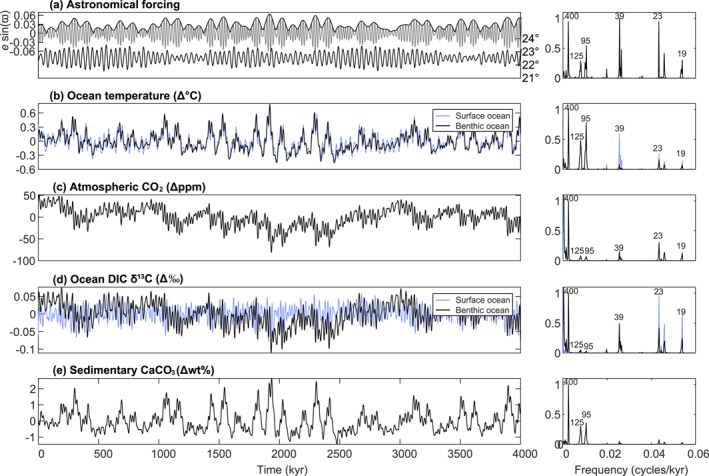
Cumulative astronomical impact of asymmetric configuration (ASYM) simulations. (a) Astronomical forcing parameters (as per Figure 3a). (b) Annual global mean temperature change in the benthic (black), and surface (blue) ocean. (c) Annual global mean *p*CO_2_ change. (d) Annual global mean δ^13^C change in the deep (black) and surface (blue) ocean dissolved inorganic carbon (DIC) reservoir. (e) Global mean sedimentary CaCO_3_ change. All variables are accompanied by their respective Fast Fourier Transforms (FFTs), normalized to the peak with maximum power. The combined impact of all four climate‐carbon feedbacks on atmospheric, ocean, and sedimentary reservoirs.

In addition to allowing us to elucidate the role of individual feedbacks, our experimental framework provides the net (Earth system) astronomical forcing response of ocean circulation, CO_2_ solubility, ocean productivity, CaCO_3_ dissolution and terrestrial weathering feedbacks combined (Figure 7 for ASYM and Figure S7 for SYMM). Under the modeled ice‐free greenhouse climate state, ocean circulation, CO_2_ solubility, and CaCO_3_‐dissolution feedbacks are positive with respect to the astronomically driven temperature change and generate an increase in the average range in atmospheric CO_2_ of ∼6 ppm (circulation and solubility) and ∼14 ppm (19 minus 3.5 ppm) (CaCO_3_ dissolution) at the long eccentricity period in ASYM. On the other hand, marine productivity reduces the average long eccentricity range in *p*CO_2_ by ∼3 ppm (6.2 minus 3.5 ppm) and terrestrial weathering increases the range, but flips the phase, equating to a change of ∼45 ppm (19 plus 26 ppm) in the long eccentricity cycle in ASYM (Table S2). Hence, marine productivity and terrestrial weathering act as negative feedbacks. The net Δ*p*CO_2_ as simulated in Exp.4 is dominated by the terrestrial weathering feedback and slightly negative on eccentricity timescales, resulting in a reduced average range in global mean benthic temperature of 0.25°C at long eccentricity periods compared to a 0.30°C average range in Exp.0 that excludes all carbon‐climate feedbacks. For SYMM, ocean circulation and solubility, and CaCO_3_ dissolution increase the 400 kyr average range in *p*CO_2_ by 4.5 and 13 ppm, respectively, whereas marine productivity decreases the range by 0.5 ppm and terrestrial weathering flips the phasing between eccentricity and *p*CO_2_, equating to a change of 41 ppm (17 plus 24 ppm).

Our framework considers two groups of feedbacks, (a) astronomically forced changes in CO_2_ solubility, ocean circulation, and marine surface productivity, which redistribute carbon between the ocean and atmosphere, and (b) astronomical forcing of carbonate compensation and silicate weathering feedbacks, which remove and add carbon to the atmosphere‐ocean system. The feedbacks related to carbon redistribution act on timescales comparable to or smaller than those of global ocean overturning and therefore respond rapidly to astronomical forcing. Redistribution of carbon contributes to high frequency cyclicity produced in atmospheric *p*CO_2_, global mean DIC δ^13^C, and global mean CaCO_3_ preservation. However, the well‐ventilated ocean simulated here, as a result of the simplified continental configurations likely results in a lower estimate of ocean circulation variability in response to astronomical forcing. For example, a more poorly ventilated ocean basin would store more carbon in the deep ocean reservoir and could release relatively more CO_2_ to the atmosphere in response to small changes in ocean overturning strength, as shown with a theoretical framework along with a 3D ocean biogeochemistry model (Kwon et al., 2011) and more specifically for the Last Glacial Maximum using the cGENIE model (Ödalen et al., 2018).

By removing and adding carbon to the atmosphere‐ocean system, carbonate compensation and silicate weathering feedbacks drive the most distinct responses in the carbon cycle, both in terms of the total variability in response to astronomical forcing and shifts in spectral power. Generally, long‐term carbon feedbacks enhance power at low frequencies at the expense of high frequencies by acting as a low‐pass filter at timescales comparable to the residence time of carbon in the ocean‐atmosphere. This occurs in both ASYM and SYMM simulations and is therefore independent of the continental configuration. The spectral signals in ASYM and SYMM of modeled *p*CO_2_, benthic DIC δ^13^C, and wt% CaCO_3_ are thus similar in the sense that the long eccentricity cycle dominates. However, a notable difference is the absence of precession cycles in SYMM (Figure S7) and their presence in ASYM (Figure S7) due to the hemispherically asymmetric impact of ocean circulation, solubility, and marine productivity on the redistribution of carbon between the surface and deep ocean in ASYM. High frequency cycles are particularly exacerbated in DIC δ^13^C of ASYM where marine productivity is greatest in the SH and local, seasonal changes in temperature and [CO_2_ (aq)] affect the carbon isotope fractionation of POC and CaCO_3_.

While these simulations provide a systematic analysis of mechanisms through which astronomical forcing impacts the climate‐carbon system, our experiments do not include a comprehensive representation of all relevant mechanisms. Our focus in this current paper is on the inorganic (carbonate) carbon cycle and feedbacks. We do not include a terrestrial biosphere (land plants and soils) or other reduced carbon reservoir (e.g., permafrost, methane hydrates). We also deliberately use the most simplistic parameterization of export productivity, so it is likely that we underestimate its sensitivity to astronomical forcing. Export productivity in these experiments is dependent only on the nutrient (phosphate) concentration with a modifier for light availability. Temperature dependence in parameterizations of export production of particulate organic matter and carbonate (Monteiro et al., 2012), and water column remineralization (Crichton et al., 2020; John et al., 2014) likely modify the carbon cycle response to astronomical forcing (see Crichton et al., 2020 for an analysis of the role of temperature in remineralization).

Moreover, we have applied a reflective boundary for organic matter and phosphate at the sediment‐water interface, neglecting organic matter burial and assume an invariant nutrient inventory. Organic matter preservation and burial processes, in addition to reflecting changes in overlying export production, are temperature and oxygen dependent (Hülse et al., 2017), implying several different pathways by which astronomical forcing may additionally impact atmospheric *p*CO_2_ and DIC δ^13^C. Furthermore, inclusion of an open system for phosphate may result in distinct astronomical fingerprints as the phosphate residence time is similar in duration to high frequency astronomical cycles (van Cappellen & Ingall, 1996).

In comparison to benthic high‐resolution paleoclimate records throughout the Cenozoic (Barnet et al., 2019; Beddow et al., 2016; Holbourn et al., 2013; Littler et al., 2014; Pälike et al., 2006; Tian et al., 2018; Westerhold et al., 2018), our simulations show minor variability in δ^13^C on astronomical timescales (the maximum variability in simulated δ^13^C cycles is less than 0.2‰ in ASYM). This model‐data discrepancy is especially pronounced as we are evaluating modeled water column δ^13^C and not considering the impact of bioturbation on the range of preserved cycles. The muted simulated δ^13^C cycles very likely reflect missing feedbacks in the organic carbon cycle. Feedbacks in the marine organic carbon cycle will be addressed systematically in a follow‐on paper.

Finally, an important caveat to our results is that, while cGENIE includes a dynamic ocean model, the 2D energy‐moisture balance atmosphere does not reflect the potential impacts of astronomical forcing on atmospheric dynamics, for instance including changing patterns of winds or cloud cover. It is unclear exactly how inclusion of these dynamics in response to astronomical forcing would modify the changes in the global mean annual air temperature (MAAT) simulated here. However, the maximum variability in MAAT without carbon feedbacks in Exp.0 (1.7°C) corresponds well with the difference in MAAT between and eccentricity maxima and minima for the Eocene with 2 × (∼1.5°C) and 4 × (∼2°C) preindustrial CO_2_ concentrations simulated with the HadCM3L GCM (Lunt et al., 2011).

### 3.7 Implications for the Interpretation of Marine Proxy Records

While simulated cycles in benthic temperature, δ^13^C, and wt% CaCO_3_ show modest ranges in comparison to proxy records of temperature (e.g., δ^18^O), carbon isotopes (δ^13^C), and CaCO_3_ from early Cenozoic ice‐free climates, we can also compare model and data in terms of the relative distribution of power and phasing in key climate and carbon cycle metrics in response to astronomical forcing. Early Cenozoic deep‐sea proxy records generally show pronounced 100 and 400 kyr eccentricity cycles (Barnet et al., 2019; Lauretano et al., 2015; Littler et al., 2014; Sexton et al., 2011; Westerhold et al., 2011; Zachos et al., 2010). Our results demonstrate that mean annual temperature spectra are dominated by eccentricity, despite the small impact of eccentricity on the insolation forcing. Strong 400 and 100 kyr power is also consistent with our modeled wt% CaCO_3_ records, but 100 kyr power is notably lacking from modeled δ^13^C.

Importantly, modeled wt% CaCO_3_ is the only model metric we report that is influenced by the low‐pass filter effect of sedimentary mixing. Thus, CaCO_3_ preservation is also dominated by 100 and 400 kyr cycles, despite the significance of precession in carbonate export and deep ocean chemistry. The spectral difference between carbonate export, deep ocean [CO_3_^2−^] and wt% CaCO_3_ is hence also likely to shift power in proxy records of temperature and δ^13^C toward longer periods. However, this process cannot create power at the shorter 100 kyr period that is currently absent from modeled water column δ^13^C. Other processes, not included here, must be invoked to produce the 100 kyr δ^13^C eccentricity cycles in proxy records.

**Figure 8:**
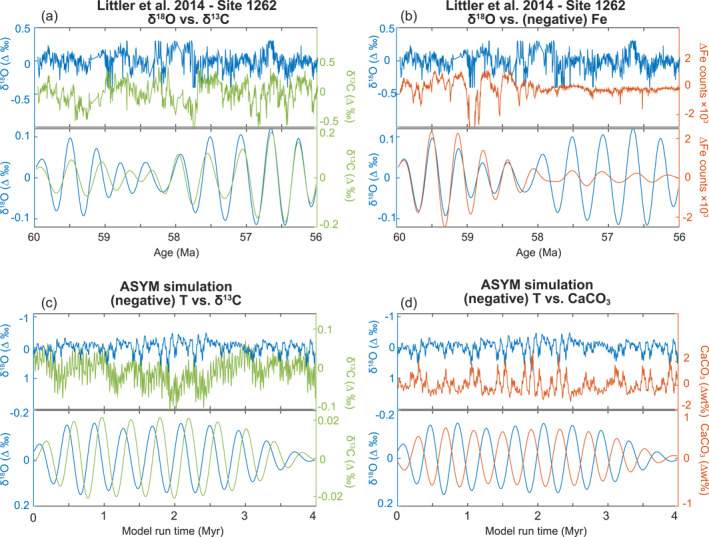
Phase analysis of paleoclimate proxies and comparable model output. (a and b) Benthic paleoenvironmental records from ODP Site 1262 of temperature (δ^18^O, in blue), stable carbon isotopes (δ^13^C, in green), and Fe counts (in orange). Below, the associated bandpass filtered signal of the long eccentricity cycle (320–480 kyr). (c and d) Simulated (negative) annual global mean deep ocean temperature (in blue), δ^13^C of dissolved inorganic carbon (DIC) (in green), and sedimentary carbonate weight percentage (in orange) of asymmetric configuration (ASYM) simulations Exp.4. Below, the associated bandpass filtered signal of the long eccentricity cycle.

Early Cenozoic benthic foraminiferal records that contain evidence for astronomically paced “hyperthermals” (∼60–48 Ma) also record characteristic phase relationships between temperature, δ^13^C, and CaCO_3_. Eccentricity‐paced temperature maxima lead δ^13^C minima by 5–20 kyr and CaCO_3_ minima often lead δ^13^C minima by ∼2–40 kyr (Littler et al., 2014; Turner, 2014; Westerhold et al., 2018). However, our model simulations including all four feedbacks (Exp.4) show significant differences in the phase relationship between temperature, global mean wt% CaCO_3_, and the δ^13^C (Figure 8). Most strikingly, global mean wt% CaCO_3_ is higher during eccentricity temperature maxima, a consequence in the model of elevated export production, warmer deep ocean temperatures, and elevated deep ocean [CO_3_^2−^] caused by both stronger ocean overturning and enhanced terrestrial weathering. A few possibilities could explain this model‐data discrepancy. First, the terrestrial weathering feedback might have been weaker than modeled here during the early Cenozoic (Caves et al., 2016; Kump & Arthur, 1997; van der Ploeg et al., 2018). Second, changes in shallow shelf carbonate burial might have compensated for deep sea burial. Changes in shelf burial might have corresponded to sea level fluctuations even in an ice‐free world (Li et al., 2018; Liu et al., 2021). Third, additional carbon cycle feedbacks (not modeled) involving the organic carbon cycle might have dominated the phase relationship between temperature and carbonate preservation.

The hypothesis for a weaker weathering feedback has been postulated across climatic warming events including the Middle Eocene Climate Optimum (van der Ploeg et al., 2018) as well as the end‐Permian (Kump, 2018). A gradual strengthening of the weathering feedback over the past 50 Myr has also been proposed to explain the long‐term decline in *p*CO_2_ across the Cenozoic and to explain why the early Cenozoic was more sensitive to sudden carbon cycle perturbations (e.g., hyperthermals) than the mid and late Cenozoic (Caves et al., 2016). If silicate weathering rates are limited by factors like the thickness of soils (Kump, 2018), rock weatherability (Caves et al., 2016; van der Ploeg et al., 2018), or rate of rock uplift (Kump and Arthur, 1997) that prevent a strong positive response to increasing temperature, then the silicate weathering feedback would not have operated as modeled here in response to eccentricity‐induced warming. Indeed, in Exp.3 with fixed terrestrial weathering, sedimentary carbonate content is relatively reduced during eccentricity‐driven temperature maxima (compared to Exp.1, 2, and 4) when the weathering flux of carbonate ions to the ocean is insufficient to sustain CaCO_3_ burial.

The second possibility is that the residual flux of the products of weathering to the open ocean were modulated by changes in shelf burial. An astronomical forcing that induces a greater shallow marine deposition response as compared to the total global weathering response, will result in the net supply to the open ocean becoming anti‐phased. This could occur at least partly in response to changes in sea level (Li et al., 2018; Liu et al., 2021) and greater shelf area due to the thermal expansion of seawater (Lyle et al., 2008; Opdyke & Wilkinson, 1988), or directly from temperature controls on coral growth. However, the neritic system is complex and we lack a model representation of shallow water carbonate burial able to account for sea‐level and accommodation space controls on neritic deposition.

Finally, the reduced carbon cycle may also play a key role in early Cenozoic carbon‐climate variability, with, for instance, massive release of ^13^C‐depleted carbon into the ocean‐atmosphere proposed to explain hyperthermal warming (e.g., Dickens, 2003; Lunt et al., 2011; DeConto et al., 2012; Komar, et al., 2013; Zachos et al., 2010; Zeebe et al., 2017). Given that our model framework currently neglects the dynamics of both terrestrial and marine reduced carbon reservoirs, we instead estimate the consequences of reduced carbon release in association with eccentricity maxima using previous cGENIE simulations without astronomical forcing. Vervoort et al., (2019) simulated 0.5‰ negative δ^13^C excursions with a duration of 100 kyr (comparable in size and duration to early Cenozoic δ^13^C cycles) via input of 600–1,500 Pg carbon with a δ^13^C signature of −22‰. The required carbon input masses resulted from the prescribed carbon input durations between 25 and 75 kyr (or a maximum rate of 0.02 Pg yr^−1^). This forcing yields an increase in global temperature of 0.5°C–1°C. Yet, these “slow” perturbations to the carbon cycle do not generate notable changes in modeled wt% CaCO_3_ since the temperature‐dependent weathering feedback increases transport of alkaline carbon species to the ocean at comparable rates (Vervoort et al., 2019). Carbon fluxes must exceed 0.2 Pg yr^−1^ to offset enhanced weathering fluxes and drive dissolution coincident with warming.

Moreover, model‐data discrepancy exists between the small 5–20 kyr lag between δ^18^O and δ^13^C in early Cenozoic proxy records, compared to the large lag of ∼100 kyr simulated in cGENIE between benthic temperature and benthic DIC δ^13^C on eccentricity timescales (Figure 8a vs. 8d, Table S2). Large modeled lags between temperature and δ^13^C are produced only in experiments including CaCO_3_ compensation and/or weathering feedbacks and occur because the small isotopic difference between inorganic carbon reservoirs requires long‐sustained imbalances to produce a noticeable effect in the isotopic signature of the deep ocean DIC reservoir. Rapid input of low δ^13^C carbon would leave a notable imprint on the DIC reservoir much more rapidly due to the larger isotopic difference. Hence, geologically rapid release of low δ^13^C carbon at eccentricity paced intervals therefore fits best with the phase relationships observed between δ^18^O, δ^13^C, and CaCO_3_ across astronomical timescales in early Cenozoic marine paleoclimate records and strongly points to the exclusion of dynamic reduced carbon reservoirs as the primary reason for model‐data mismatch.

## 4 Conclusion

We have simulated the transient impact of astronomical forcing on the climate and (inorganic) carbon cycle using a dynamic 3D carbon‐enabled Earth system model under ice‐free conditions. Global annual mean temperature is dominated by eccentricity power with cycles of 1.3°C–1.7°C, depending on the continental distribution. While eccentricity also dominates power spectra of early Cenozoic benthic foraminiferal δ^18^O records, observed variations correspond to cycles of 2°C–3°C in deep ocean temperature, suggesting that additional feedbacks, not modeled here, roughly doubled the temperature response to insolation forcing.

We focused in this paper on feedbacks involving CO_2_ solubility, marine productivity, CaCO_3_ sedimentation, and terrestrial weathering. While these do not significantly amplify modeled temperature variability, they do cause distinct shifts in modeled carbon cycle proxies. The CO_2_ solubility feedback, ocean circulation, and marine primary production redistribute carbon between the atmosphere, surface, and deep ocean, producing high frequency (precession and obliquity) spectral signals in the benthic DIC δ^13^C reservoir. Long‐sustained imbalances between CaCO_3_ sedimentation and terrestrial weathering dampen high frequency variability in δ^13^C and shift power spectra toward long eccentricity. Global mean wt% CaCO_3_ increases during eccentricity‐driven warm intervals when enhanced weathering elevates ocean ALK. The opposite relationship between temperature and wt% CaCO_3_ in early Cenozoic records implies that the strength of the terrestrial weathering feedback was reduced, a shift in shallow shelf versus deep carbonate burial, and/or carbon cycle dynamics were controlled by additional (organic) carbon feedbacks. Geologically rapid, short‐lived fluxes of reduced carbon into the atmosphere‐ocean reservoir at eccentricity maxima are likely necessary to reconcile model‐data discrepancies.

## Supporting information

Supporting Information S1

## Data Availability

The version of the “muffin” release of the cGENIE Earth system model code used in this paper is tagged as release v0.9.25 and has is assigned a https://doi.org/10.5281/zenodo.5130666. Configuration files for the specific experiments presented here can be found in the directory: genie‐userconfigs/MS/vervoortetal.2021. Details on the experiments, plus the command line needed to run each one, are given in the README.txt file in that directory. All other configuration files and boundary conditions are provided as part of the code release. A manual detailing code installation, basic model configuration, tutorials covering various aspects of model configuration and experimental design, plus results output and processing, is assigned a https://doi.org/10.5281/zenodo.5130672.
