# Supplementary material for: Earth System Model Analysis of How Astronomical Forcing Is Imprinted Onto the Marine Geological Record: The Role of the Inorganic (Carbonate) Carbon Cycle and Feedbacks
Source: Paleoceanogr Paleoclimatol. 2021 Sep 30;36(10):e2020PA004090. doi: 10.1029/2020PA004090 (PMC9293320; doi:10.1029/2020PA004090)
Supplement: Supplementary file 1 — Supporting Information S1 [file PALO-36-0-s001.pdf]

Paleoceanography and Paleoclimatology

Supporting Information for

Earth System Model Analysis of how Astronomical Forcing is Imprinted onto the Marine Geological Record: The role of the Inorganic (Carbonate) Carbon Cycle and Feedbacks

P. Vervoort[1], S. Kirtland Turner[1], F. Rochholz[2†], and A. Ridgwell[1]

[1] Department of Earth and Planetary Science, University of California, Riverside, Riverside, CA, USA
[2] MARUM Center for Marine and Environmental Sciences, University of Bremen, Bremen, Germany

[†] Currently at Research Group for Earth Observation, Department of Geography, Heidelberg University of Education, Heidelberg, Germany

Contents of this file

Table S1. cGENIE spin up parameters and steady state output for SYMM and ASYM simulations

Table S2. Average ranges of astronomical cycles and phasing relative to eccentricity forcing for benthic temperature, *p*CO2, δ^{13}CDIC, and $CaCO_3$ wt% for Exp.0-4

Figure S1. Ocean sediment depth distribution for SYMM and ASYM

Figure S2. Astronomical climate evolution in SYMM simulation (Exp.0)

Figure S3. Astronomical response of the ocean circulation and solubility feedback in SYMM (Exp.1)

Figure S4. Astronomical response of the marine productivity feedback in SYMM (Exp.2)

Figure S5. Astronomical response of the calcium carbonate sedimentation feedback in SYMM (Exp.3)

Figure S6. Astronomical response of the terrestrial weathering feedback in SYMM (Exp.4)

Figure S7. Cumulative impact of astronomical forcing of all carbon-climate feedbacks in SYMM

Figure S8. Relative spectral power of astronomical elements in pCO_2, δ^{13}CDIC, and $CaCO_3$ wt%

Figure S9. Astronomical change in benthic $[CO_3^{2-}]$ for ASYM and SYMM (Exp.1)

Table S1. Input parameters for carbonate (Ca) weathering, silicate (Si) weathering, volcanic outgassing, and the reference temperature for weathering for the initial spin-up state of SYMM and ASYM simulations. Below, the steady state model output values for atmospheric carbon dioxide ($_{p}CO_2$) concentrations, global annual mean atmospheric temperature, surface and deep ocean temperature, total amount of ocean alkalinity (ALK) and dissolved inorganic carbon (DIC), and global mean calcium carbonate ($CaCO_3$) in marine sediment.

**Parameters**	**SYMM**	**ASYM**
Ca weathering flux	7.00 Tmol C yr^{-1}	7.00 Tmol C yr^{-1}
Si weathering flux	6.93 Tmol C yr^{-1}	5.60 Tmol C yr^{-1}
Volcanic outgassing	7.00 Tmol C yr^{-1}	7.00 Tmol C yr^{-1}
Reference temperature	23.79°C	19.45°C
**Results**	**SYMM**	**ASYM**
Atmospheric pCO_2	834 ppm	828 ppm
Atmospheric temperature	22.6°C	23.1°C
Surface/deep ocean temperature	24.5°C / 6.6°C	27.2°C / 7.8°C
Ocean ALK	1629 μmol kg^{-1}	1517 μmol kg^{-1}
Ocean DIC	1661 μmol kg^{-1}	1557 μmol kg^{-1}
Sedimentary $CaCO_3$	51 wt%	47 wt%

Table S2. The average ranges of individual astronomical cycles in the global annual mean benthic temperature (ben T), atmospheric CO_2, benthic DIC $\delta^{13}C$, and wt% $CaCO_3$ across the modeled four million years. Average ranges are calculated from the FFT amplitude spectrum. Below, the relative phasing (in degrees) between the imposed short and long eccentricity forcing and the proxies. Positive values indicate that eccentricity forcing is leading. Negative values indicate that the proxy leads eccentricity. Green shows relatively in-phase relationships. Red colors suggest a relatively anti-phased relationship.

	ASYM					SYMM				
	Exp.0	Exp.1	Exp.2	Exp.3	Exp.4	Exp.0	Exp.1	Exp.2	Exp.3	Exp.4
Average range (=amplitude*2)										
ben T, precession	0.07°C	0.05°C	0.06°C	0.06°C	0.11°C	--	--	--	--	--
ben T, obliquity	--	0.08°	--	--	0.08°C	0.06°C	0.06°	0.05°C	0.06°C	0.07°
ben T, short ecc	0.25°C	0.28°C	0.26°C	0.30°C	**0.25°**	0.22°C	0.24°C	0.23°C	0.27°C	0.22°C
ben T, long ecc	**0.30°C**	**0.33°C**	**0.31°C**	**0.39°C**	0.25°C	**0.26°C**	**0.28°C**	**0.28°C**	**0.34°C**	**0.23°C**
CO_2, precession	2.2 ppm	2.4 ppm	**5.5 ppm**	3.9 ppm	17 ppm	2.6 ppm	2.8 ppm	1.4 ppm	2.7 ppm	3.1 ppm
CO_2, obliquity	4.8 ppm	5.2 ppm	1.9 ppm	--	11 ppm	--	--	2.4 ppm	2.4 ppm	5.9 ppm
CO_2, short ecc	4.8 ppm	5.2 ppm	2.9 ppm	12 ppm	9 ppm	3.6 ppm	3.8 ppm	3.4 ppm	12 ppm	7.2 ppm
CO_2, long ecc	**5.6 ppm**	**6.2 ppm**	3.5 ppm	**19 ppm**	**26 ppm**	**4.2 ppm**	**4.5 ppm**	**4.0 ppm**	**17 ppm**	**24 ppm**
$d^{13}C$, precession	0.016 ‰	0.016 ‰	0.021 ‰	0.021 ‰	0.028 ‰	**0.012 ‰**	**0.012 ‰**	**0.013 ‰**	**0.013 ‰**	0.013 ‰
$d^{13}C$, obliquity	**0.024 ‰**	**0.024 ‰**	**0.025 ‰**	**0.022 ‰**	0.028 ‰	0.003 ‰	--	0.005 ‰	0.005 ‰	0.005 ‰
$d^{13}C$, short ecc	0.005 ‰	0.005 ‰	0.005 ‰	0.009 ‰	0.009 ‰	0.003 ‰	0.003 ‰	0.003 ‰	0.003 ‰	0.008 ‰
$d^{13}C$, long ecc	0.005 ‰	0.006 ‰	0.006 ‰	0.011 ‰	**0.036 ‰**	0.002 ‰	0.002 ‰	0.002 ‰	0.003 ‰	**0.032 ‰**
$CaCO_3$, precession	0.3 wt%	0.3 wt%	0.1 wt%	0.1 wt%	0.4 wt%	--	--	0.2 wt%	0.2 wt%	0.2 wt%
$CaCO_3$, obliquity	0.4 wt%	0.4 wt%	0.1 wt%	0.1 wt%	0.3 wt%	0.1 wt%	0.1 wt%	--	0.1 wt%	0.2 wt%
$CaCO_3$, short ecc	0.3 wt%	0.3 w%	0.5 wt%	**0.4 wt%**	0.7 wt%	0.2 wt%	0.6 wt%	0.6 wt%	**0.4 wt%**	0.8 wt%
$CaCO_3$, long ecc	**0.4 wt%**	**0.5 wt%**	**0.7 wt%**	0.2 wt%	**1.1 wt%**	**0.3 wt%**	**0.8 wt%**	**0.9 wt%**	0.2 wt%	**1.1 wt%**
Relative phasing (multi-taper coherence) [values in degrees]										
short ecc - ben T	4	5	4	11	-9	4	4	4	10	-8
short ecc - CO_2	6	7	6	36	-112	10	11	9	29	-96
short ecc - $d^{13}C$	29	29	29	33	-82	132	132	136	108	-126
short ecc - $CaCO_3$	29	29	31	-18	37	25	26	26	-17	31
long ecc - ben T	1	1	1	4	-30	1	1	1	3	-30
long ecc - CO_2	2	2	1	10	-125	2	2	2	8	-118
long ecc - $d^{13}C$	7	8	7	-22	-117	164	163	166	-110	-123
long ecc - $CaCO_3$	6	7	8	-50	-9	6	6	7	-40	-11

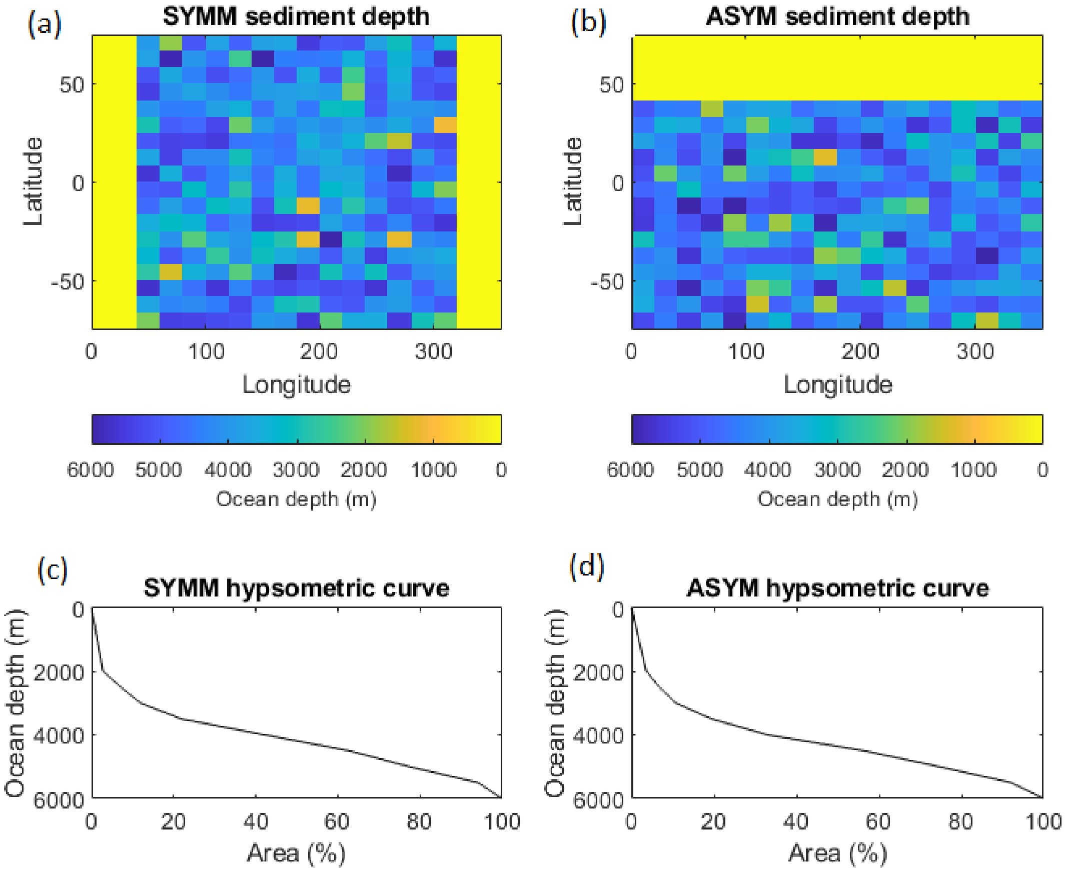

Figure S1. Ocean depth of SYMM and ASYM. A latitude-longitude ocean sediment depth grid for (a) SYMM and (b) ASYM. Bright yellow colors indicate land masses. The hypsometric curve for (c) SYMM and (d) ASYM ocean sediments showing the cumulative distribution of ocean sediment depth generated with 'muffingen' v0.9.25 (DOI: 10.5281/zenodo.5130677).

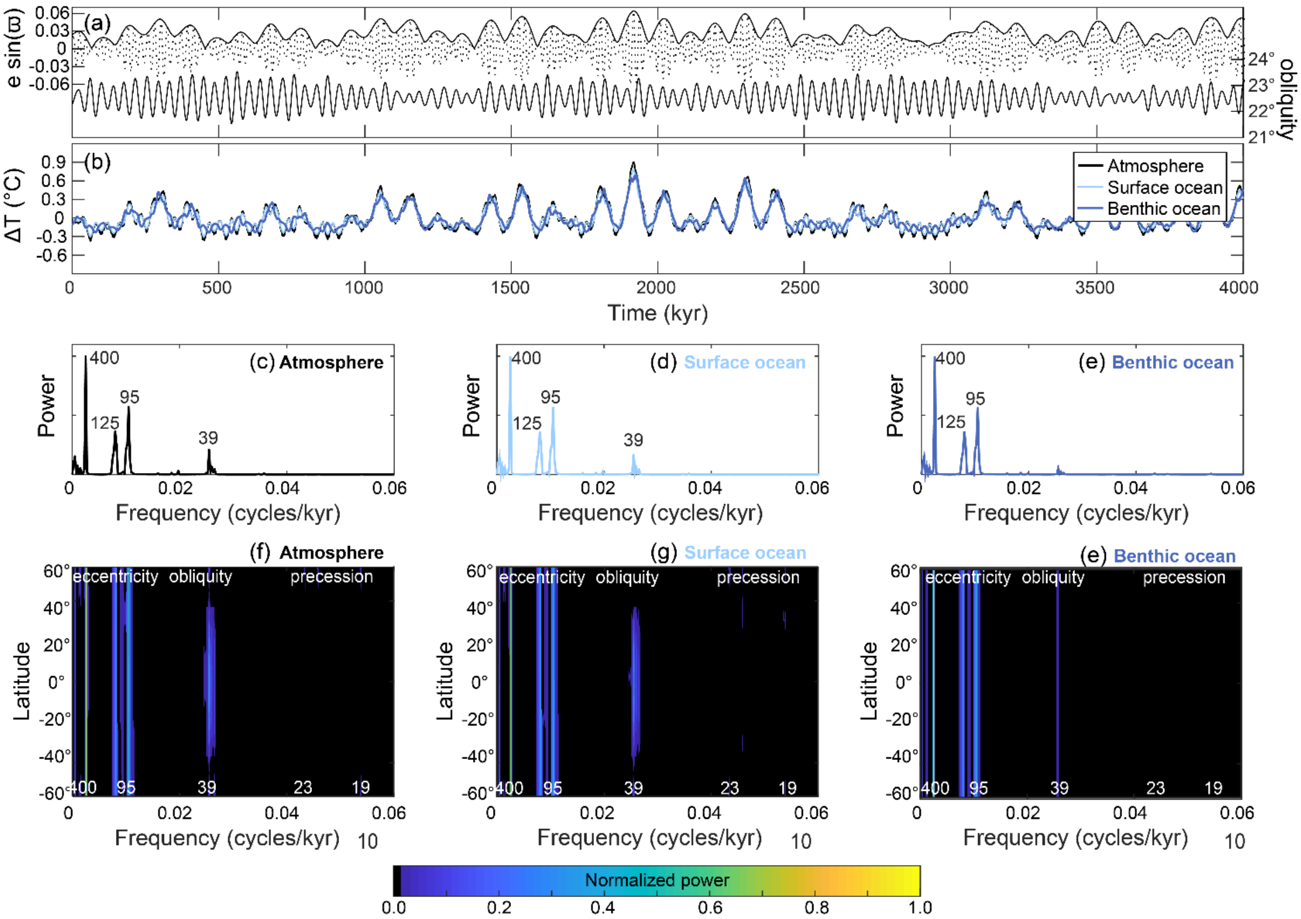

Figure S2. Astronomical climate evolution in SYMM of Exp.0. (a) The eccentricity, obliquity, and the precession index (e sin ϖ) used to calculate the daily mean insolation across four million years of cGENIE simulation. (b) The simulated change in the global annual mean temperature in the atmosphere (black), surface (light blue), and deep ocean (dark blue) with their respective normalized power spectra in panels (c-e) calculated using the Fast Fourier Transform. Each value near a spectral peak indicates the associated period of the cycle in kyr. The power spectra of temperature in the (f) atmosphere, (g) surface, and (h) deep ocean are shown for each latitude.

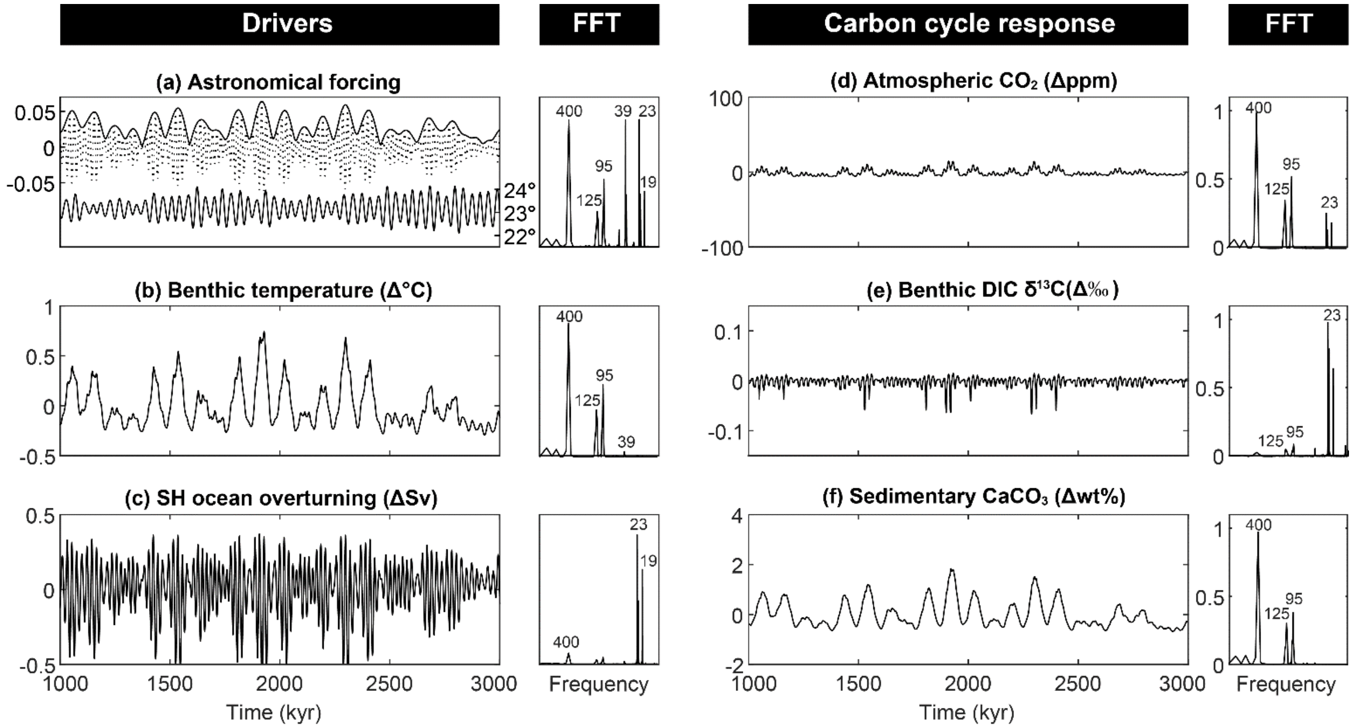

Figure S3. Exp.1, ocean circulation and CO_2 solubility feedbacks in SYMM. (a) Astronomical forcing parameters (eccentricity, obliquity (in degrees), and precession index (e sin ϖ)) and their Fast Fourier Transform (FFT) normalized to the highest individual power. (b) Change in benthic ocean temperature. (c) Change in the maximum Southern Hemisphere ocean overturning strength, defined by the maximum overturning strength reached in the SH across all ocean depth levels. (d) Change in atmospheric CO_2. (e) Change in benthic DIC $\delta^{13}C$. (f) Change in wt% $CaCO_3$. All variables are annual global mean values and are accompanied by their respective FFTs, normalized to the peak with maximum power.

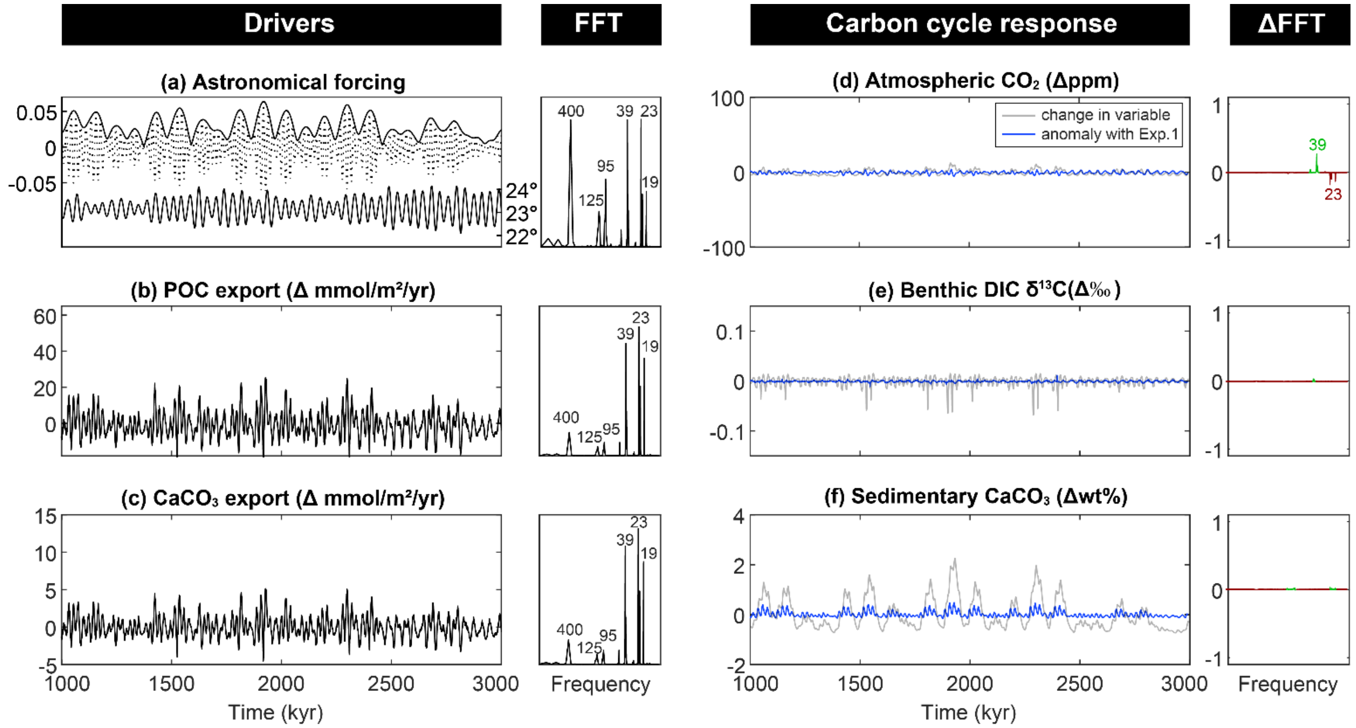

Figure S4. Exp.2, marine surface productivity feedback in SYMM. (a) Astronomical forcing parameters (as per Figure 3a). (b) Change in the export of particulate organic carbon (POC). (c) Change in the export of $CaCO_3$. (d) Change in atmospheric CO_2. (e) Change in benthic DIC $\delta^{13}C$. (f) Change in wt% $CaCO_3$. All variables are annual global mean values and are accompanied by their respective FFTs, normalized to the peak with maximum power. In blue, the values are plotted as anomalies with the previous experiment and depict the change driven by the marine productivity feedback only. Likewise, the FFTs are plotted as anomalies. A positive (green) value on the y-axis indicates an increase in relative power and a negative (red) value indicates a decrease in relative power compared to the previous experiment.

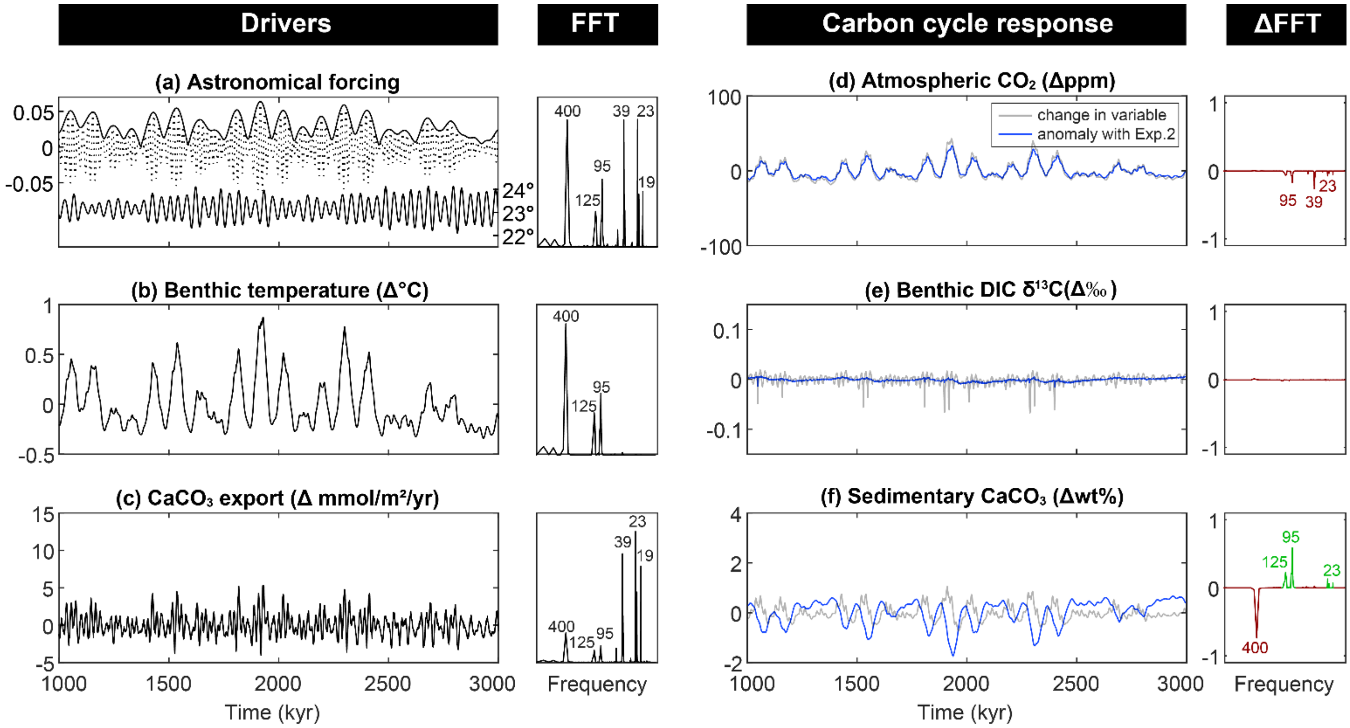

Figure S5. Exp.3, deep marine $CaCO_3$ feedback in SYMM. (a) Astronomical forcing parameters (as per Figure 3a). (b) Change in benthic ocean temperature. (c) Change in the export of $CaCO_3$. (d) Change in atmospheric CO_2. (e) Change in benthic DIC $\delta^{13}C$. (f) Change in wt% $CaCO_3$. All variables are annual global mean values and are accompanied by their respective FFTs, normalized to the peak with maximum power. In blue, the values are plotted as anomalies with the previous experiment and depict the change driven by the marine $CaCO_3$ feedback only. Likewise, the FFTs are plotted as anomalies. A positive (green) value on the y-axis indicates an increase in relative power and a negative (red) value indicates a decrease in relative power compared to the previous experiment.

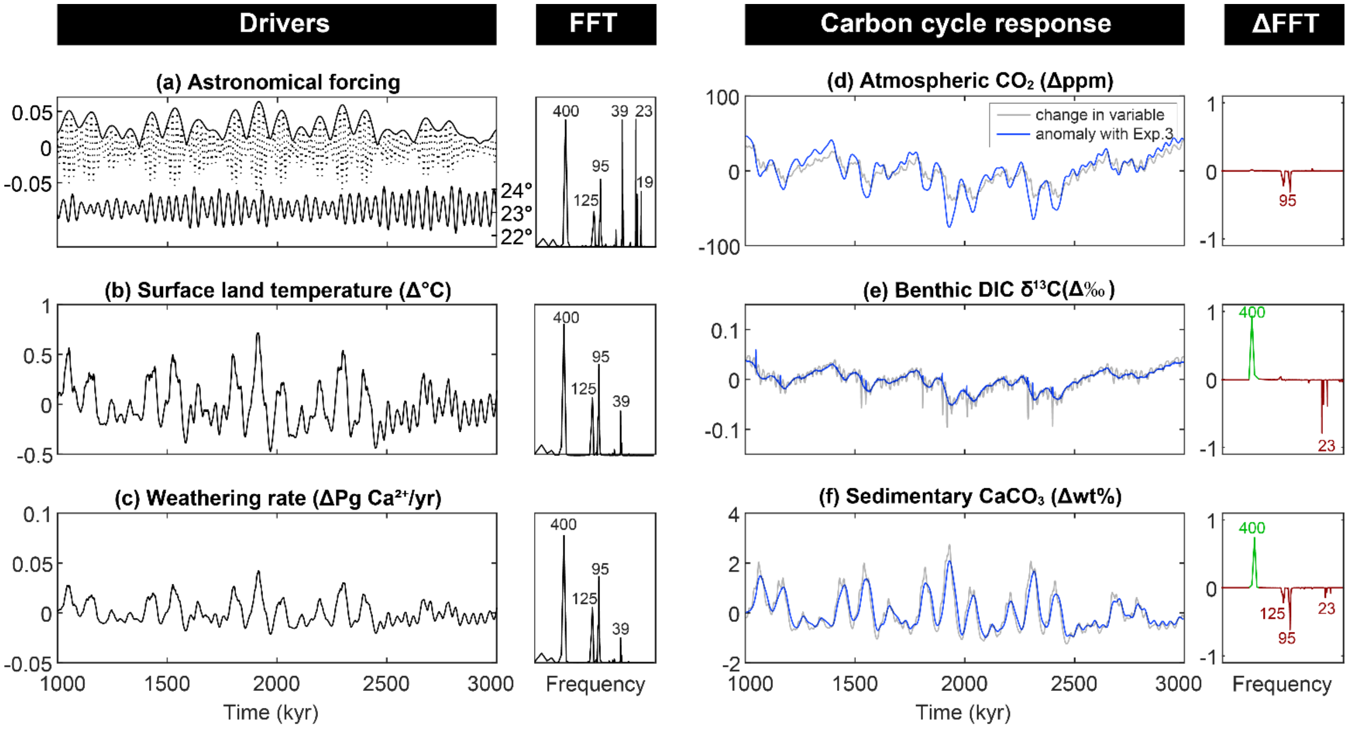

Figure S6. Exp.4, terrestrial rock weathering feedback in SYMM. (a) Astronomical forcing parameters (as per Figure 3a). (b) Change in surface land temperature. (c) Change in the rate of terrestrial weathering. (d) Change in atmospheric CO_2. (e) Change in benthic DIC $\delta^{13}C$. (f) Change in wt% $CaCO_3$. All variables are annual global mean values and are accompanied by their respective FFTs, normalized to the peak with maximum power. In blue, the values are plotted as anomalies with the previous experiment and depict the change driven by the terrestrial weathering feedback only. Likewise, the FFTs are plotted as anomalies. A positive (green) value on the y-axis indicates an increase in relative power and a negative (red) value indicates a decrease in relative power compared to the previous experiment.

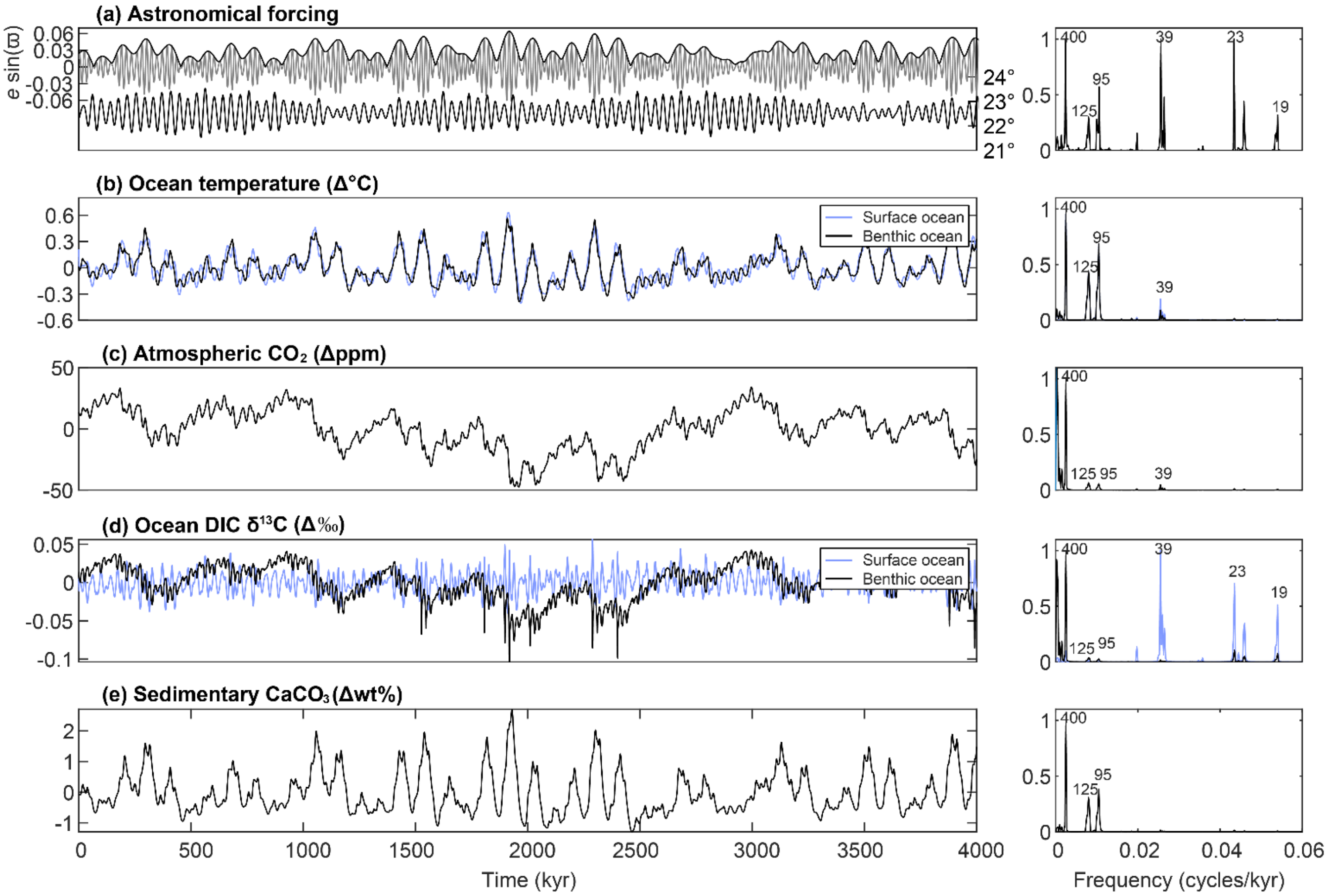

Figure S7. Cumulative astronomical impact of SYMM simulations. (a) Astronomical forcing parameters (as per Figure 3a). (b) Annual global mean temperature change in the benthic (black) and surface (blue) ocean. (c) Annual global mean pCO_2 change. (d) Annual global mean DIC $\delta^{13}C$ change in the benthic (black) and surface (blue) ocean. (e) Global mean sedimentary $CaCO_3$ change. All variables are accompanied by their respective FFTs, normalized to the peak with maximum power. The combined impact of all four climate-carbon feedbacks on atmospheric, ocean, and sedimentary reservoirs.

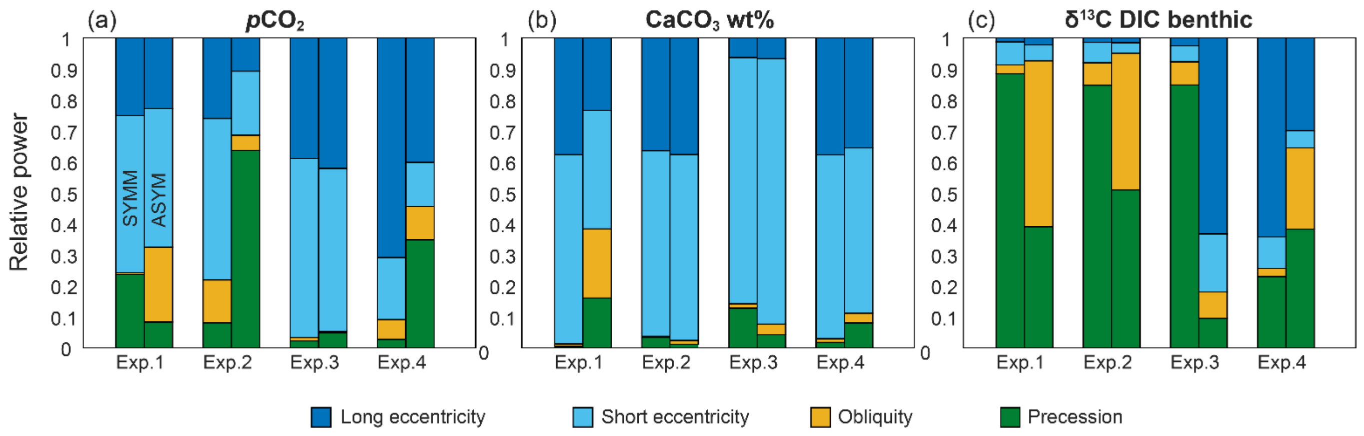

Figure S8. Relative spectral power of astronomical elements. The relative spectral power of the long (267-500 kyr) and short (78-154 kyr) eccentricity, obliquity (36-41 kyr), and precession (18-24 kyr) cycles in the simulated (a) pCO_2, (b) marine $CaCO_3$, and (c) DIC $\delta^{13}C$ in the four experimental designs of Exp.1, Exp.2, Exp.3, and Exp.4. Left bars are results from the SYMM simulations and right bars are ASYM results.

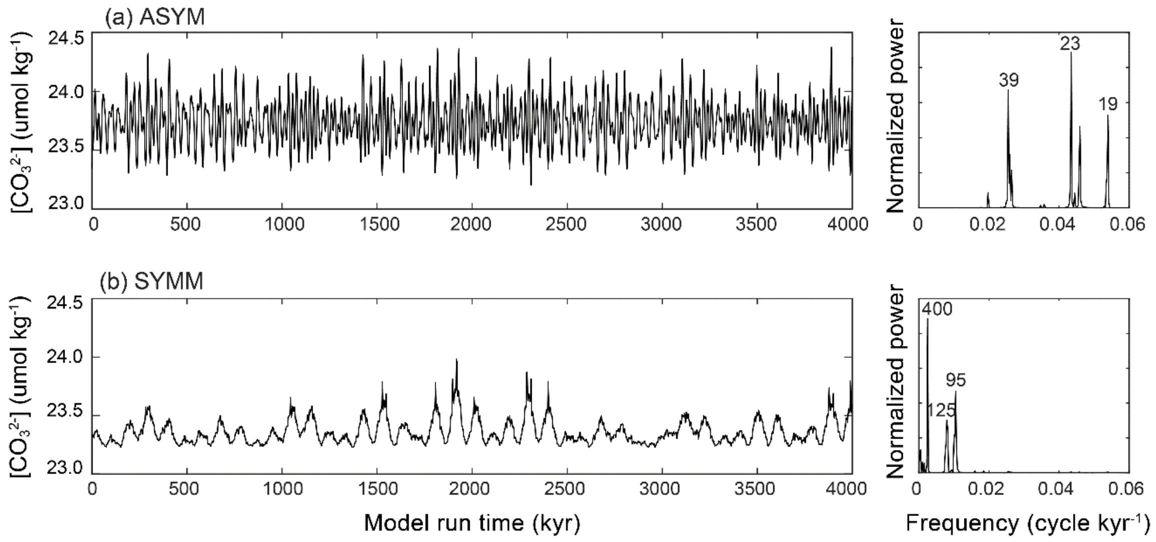

Figure S9. Benthic carbonate ion concentrations. The change in carbonate ion concentration ($[CO_3^{2-}]$) as a result of astronomically induced changes in ocean temperature, circulation, and ocean solubility (Exp.1) for (a) ASYM and (b) SYMM with their respective FFT power spectra normalized to the peak with maximum power.
